# Global Assessment of Schistosomiasis Control Over the Past Century Shows Targeting the Snail Intermediate Host Works Best

**DOI:** 10.1371/journal.pntd.0004794

**Published:** 2016-07-21

**Authors:** Susanne H. Sokolow, Chelsea L. Wood, Isabel J. Jones, Scott J. Swartz, Melina Lopez, Michael H. Hsieh, Kevin D. Lafferty, Armand M. Kuris, Chloe Rickards, Giulio A. De Leo

**Affiliations:** 1 Hopkins Marine Station, Stanford University, Pacific Grove, California, United States of America; 2 Marine Science Institute, and Department of Ecology, Evolution and Marine Biology, University of California, Santa Barbara, Santa Barbara, California, United States of America; 3 Michigan Society of Fellows, University of Michigan, Ann Arbor, Michigan, United States of America; 4 Department of Ecology and Evolutionary Biology, University of Michigan, Ann Arbor, Michigan, United States of America; 5 Children's National Health System, Washington, D.C., United States of America; 6 The George Washington University, Washington, D.C., United States of America; 7 Biomedical Research Institute, Rockville, Maryland, United States of America; 8 Western Ecological Research Center, U.S. Geological Survey, Santa Barbara, California, United States of America; 9 Department of Bioengineering, Stanford University, Stanford, California, United States of America; Center for Discovery and Innovation in Parasitic Diseases, UNITED STATES

## Abstract

**Background:**

Despite control efforts, human schistosomiasis remains prevalent throughout Africa, Asia, and South America. The global schistosomiasis burden has changed little since the new anthelmintic drug, praziquantel, promised widespread control.

**Methodology:**

We evaluated large-scale schistosomiasis control attempts over the past century and across the globe by identifying factors that predict control program success: snail control (e.g., molluscicides or biological control), mass drug administrations (MDA) with praziquantel, or a combined strategy using both. For data, we compiled historical information on control tactics and their quantitative outcomes for all 83 countries and territories in which: (i) schistosomiasis was allegedly endemic during the 20^th^ century, and (ii) schistosomiasis remains endemic, or (iii) schistosomiasis has been "eliminated," or is "no longer endemic," or transmission has been interrupted.

**Principal Findings:**

Widespread snail control reduced prevalence by 92 ± 5% (N = 19) vs. 37 ± 7% (N = 29) for programs using little or no snail control. In addition, ecological, economic, and political factors contributed to schistosomiasis elimination. For instance, snail control was most common and widespread in wealthier countries and when control began earlier in the 20^th^ century.

**Conclusions/Significance:**

Snail control has been the most effective way to reduce schistosomiasis prevalence. Despite evidence that snail control leads to long-term disease reduction and elimination, most current schistosomiasis control efforts emphasize MDA using praziquantel over snail control. Combining drug-based control programs with affordable snail control seems the best strategy for eliminating schistosomiasis.

## Author Summary

Schistosomiasis is a parasitic disease infecting more than 250 million people worldwide, with almost 800 million at risk. Over the past century, nations undertook schistosomiasis control programs, with outcomes varying from little effect to elimination. The biggest hope for elimination began about 40 years ago with the discovery of the antischistosomal drug praziquantel, after which snail control was seen as old fashioned. Here, we review control program outcomes over the past 100 years across all major schistosomiasis endemic zones, including Africa, Asia, and the Americas. We screened for differences in long-term schistosomiasis reductions among countries and found the most successful programs focused on transmission control (most often snail control, with or without engineering interventions), sometimes in tandem with praziquantel. Although praziquantel has important human-health benefits, our results suggest old-fashioned snail control has been the key to schistosomiasis elimination.

## Introduction

Can we do better at controlling schistosomiasis? Despite effective drug treatment options and large-scale drug distribution programs, most endemic areas have not yet achieved satisfactory schistosomiasis control. Today, schistosomiasis remains prevalent in Africa, Asia, and South America where trends over time forecast perpetual endemicity. Sometimes, endemicity has been because poverty constrains control efforts; otherwise, endemicity is due to failed or ineffective control attempts. With more than 250 million people still infected and elimination stalled [1,2], the World Health Assembly (WHA) called for researching and applying complementary, non-pharmaceutical control strategies for eliminating schistosomiasis in its 2012 resolution 65.21 [3]. This resolution ignited debate over the best strategies for eliminating schistosomiasis [4–9]. To add quantitative data to this debate, we evaluated schistosomiasis control strategies over the past century and screened for factors associated with elimination or long-term prevalence reductions.

Human schistosomiasis occurs where aquatic (or amphibious) intermediate host snails shed infective *Schistosoma* spp. cercariae that penetrate human skin upon contact. Infected humans suffer from anemia, stunted growth, cognitive impairment, fatigue, infertility, and sometimes, liver fibrosis or bladder cancer [10]. Most affected people live in poverty where there are few resources for research and control [11].

**Fig 1:**
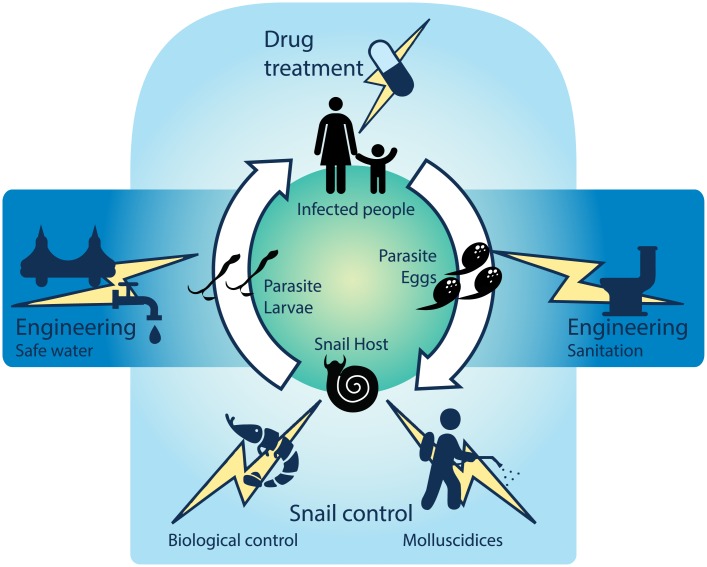
Control strategies used against schistosomiasis during the past century.

Schistosomiasis control efforts aim to disrupt the parasite’s complex life cycle (Fig 1): sanitation stops parasite eggs in urine or feces from moving into aquatic snail habitats; snail control reduces intermediate host density (parasite larvae reproduce asexually in snails); education (or “information education and communication”; IEC) helps people avoid high-risk water contacts and know when to seek treatment; and drugs—given as mass drug administrations (MDA), targeted treatment campaigns (“test-and-treat” or TAT), or through health services—kill the adult worm in the human host, with immediate and long-term health benefits for infected individuals [12]. Such efforts seem simple, but implementation often fails for economic or political reasons.

**Fig 2:**
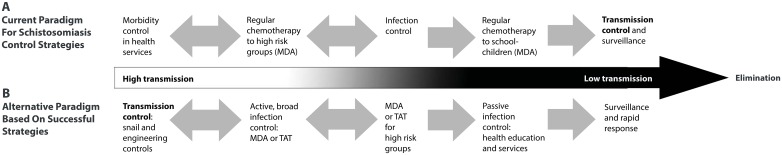
Strategy details for schistosomiasis control: the current paradigm and an alternative based on past successes. (A) The current paradigm for global schistosomiasis control, adapted from [30], and (B) an alternative strategy based on historical successes (this paper). MDA = mass drug administration, TAT = targeted (“test-and-treat”) chemotherapy.

Consensus on the “best” schistosomiasis control strategy has varied over the past century. Early Egyptian control efforts around the turn of the 20^th^ century treated human infection, then shifted toward borehole latrines as a sanitary measure in the 1930s, but with little success [13]. In the 1940s the focus shifted again, this time toward snail control using copper sulfate [13]. Control strategy recommendations by the World Health Organization (WHO) then emphasized integrated control measures, including clean water access, sanitation, snail control, health education, and health services, in addition to drug treatments [12]. In the late 1970s and early 1980s –after praziquantel became the drug-of-choice for treating schistosomiasis [14]–the integrated approach was often supplanted by chemotherapy via MDA. Praziquantel is safe and effective against adult schistosomes [15], but is ineffective against juvenile worms [16–18], and drug treatment does not prevent reinfection [19–23]. Nonetheless, large-scale research projects and influential modeling results suggested that widespread drug treatment (best when coupled with sanitation measures) would reduce schistosomiasis more than other interventions [24–27]. MDA increased in the 1990s after generic (inexpensive) praziquantel became available [15,28]. Thus, in 2001, the WHA endorsed preventive chemotherapy as the primary strategy to control schistosomiasis through reducing morbidity associated with high worm burdens [29]. Specifically, Engels et al. [30] summarized the modern, WHO-recommended schistosomiasis global control strategy as MDA in high-transmission areas to reduce morbidity and transmission reduction in low-transmission areas (Fig 2a).

Although MDA is now the most popular control strategy, some have argued that snail control is more effective [5,6,31–33]. However, a Center for Global Development working group focused on successful global health interventions points out: “We don’t know enough about what’s worked because scaled up programs are rarely evaluated systematically” [34]. Here, using objective criteria and a quantitative analysis to test for commonalities among successful control programs, we find snail control has been effective at reducing schistosomiasis.

## Methods

### Defining Success

We evaluated control programs for all areas around the world with active (autochthonous) human schistosomiasis transmission at some time in the 20^th^ century. We considered countries with little to no control effort as having *minimal control*. However if minimal control corresponded to a loss of schistosomiasis, we defined the outcome as *fortuitous elimination*. We defined control as *not (yet) successful* where (i) control has been incomplete, (ii) transmission continues, or (iii) the disease has been almost, but not yet, eliminated. We defined control as *successful* for active programs that were reported to have stopped local transmission (i.e., elimination or becoming “non-endemic”) in WHO reports or peer-reviewed assessments (e.g., [9,35]). “Elimination” implies reducing disease incidence to zero in a particular area [30,36]. There is some inconsistency in the literature on the term “eradication,” which often refers to global disease extirpation [37]. With respect to schistosomiasis, this has been applied to the regional elimination achieved by Japan. Here, because elimination, “eradication,” and non-endemicity all imply no local transmission, we treated these designations as *successful*.

### Countries and Territories Evaluated

Our goal was to evaluate control success in all countries and territories with endemic schistosomiasis during the 20^th^ century. We began with the nine countries often cited as “success stories” for schistosomiasis elimination: Iran, Japan, Lebanon, Malaysia, Martinique, Montserrat, Thailand, Tunisia, and Turkey [38]. Antigua, Jordan, and Morocco were three other potential “successes” [35,39]. We were also interested in countries that achieved great reductions in schistosomiasis prevalence including Brazil, China, the Philippines, and Egypt. Additional literature searches focused on characterizing disease and control history for all additional countries with: (i) historical disease data and (ii) recorded national- or territory-level schistosomiasis control programs. Although we found relevant data for most countries, data were contradictory for several Caribbean islands such as Guadeloupe and Dominican Republic, with some reports indicating elimination and others claiming ongoing risk. We considered these countries to be “not (yet) successful.” For several in-conflict countries such as Chad and Syria, current schistosomiasis prevalence is “unknown”, with the potential for conflict and political unrest to hinder control [40].

### Data Collection

We obtained country-specific data for several categories (S1 Table) by reading peer-reviewed published sources as well as non-peer-reviewed reports accessed through online (or hard copy) repositories, including PubMed, ISI Web of Science, Google Scholar, WHO, United Nations (UN), World Bank, United States Agency for International Development, and the UN Food and Agriculture Organization (see S1 Appendix for complete reference list). From these sources, we assessed 77 countries and six semi-autonomous territories (including Western Sahara in northern Africa, Guadeloupe, Martinique, Montserrat, Puerto Rico, and Zanzibar).

For each country, we collected information on schistosomiasis, control efforts, parasite life cycles, environmental factors, and economics. We focused on variables related to national schistosomiasis data (country- or territory-wide prevalence, infected population size, at-risk population size) and details about the control strategies implemented and their time-course. We also recorded snail and schistosome species present; island or mainland geography; and per-capita gross domestic product (GDP) in 2013 and in all years for which schistosomiasis disease data were available in each country. Further, we noted site-specific factors that might alter disease outcomes or resources for control activities (S1 Table). We limited the prevalence information, in almost every case, to country-level (or territory-level) statistics. Only for Japan, where finer scale data were available over many years, did we use large-scale and long-term regional data to assess trends, and we included only the data from the largest endemic area (the Kofu basin) in the statistical analyses. We were careful to avoid small-scale, focal studies on prevalence that might not represent the whole country. We treated countries/territories as replicates in statistical analyses done in JMP Pro version 12 [41] and R version 3.1.2 [42].

### Statistics

To test the general hypothesis that control programs can eliminate schistosomiasis, we assessed whether schistosomiasis was eliminated/non-endemic using a logistic regression, with five predictors for 68 countries/territories (excluding 15 that lacked enough data or were designated non-endemic to begin with): (i) the presence/absence of a national- or territory-level control program; (ii) status as a mainland or island (because it should be easier to achieve elimination with more isolation); (iii) the total human population infected with schistosomiasis at baseline, or before control began (because it might be harder to eliminate schistosomiasis when the starting infected population is large); (iv) the current fraction of people with access to improved water sources (as a proxy for contemporary water, sanitation, and hygiene conditions, World Bank Development Indicators, 2012 [43]); and (v) contemporary per-capita GDP (as a proxy for “wealth” status, World Bank Development Indicators, 2013 [44]; S2 Table). Using 68 countries gave us considerable statistical power to evaluate when and where control has been effective in eliminating schistosomiasis.

**Fig 3:**
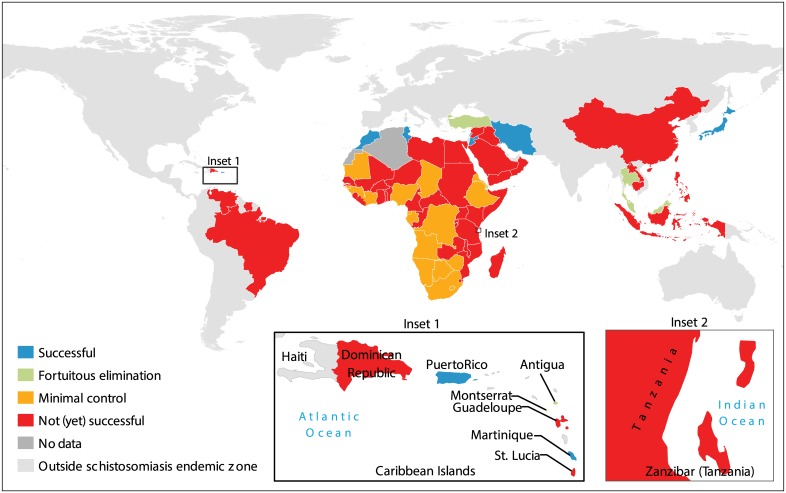
Countries and territories evaluated and their outcomes for schistosomiasis control or elimination. “Successful” = there was a control program that preceded elimination or non-endemic status; “Fortuitous elimination” = elimination or non-endemic status with no control program; “Minimal control” = endemic disease in the face of minimal to no control, even if there were some pilot or small-scale programs; “Not (yet) successful” = endemic disease with a past or present control program.

We next compared how well different control strategies reduced disease. The strategies used in historical schistosomiasis control efforts were categorized as: MDA, snail control, or engineering interventions (e.g., sanitation infrastructure, cement lined canals, drained wetlands). Each control category was further sorted according to our best estimate (based on qualitative descriptions, or sometimes, quantitative reports): *extensive/complete* (>70% of the population/area in need received treatment), *intermediate* (>30%), or *focal to none* (<30%). Although disease can be measured as intensity [45,46] (as indicated by patient egg output), there was not enough published national-level data to assess intensity means and variances. Therefore, we compared disease prevalence on a continuous scale (0 to 100% based on the schistosomiasis national prevalence at each available time point for each country/territory). We included only countries with national control programs and enough longitudinal disease data. We excluded those countries/territories with no coordinated control effort (“Minimal control” in Table 1, Fig 3). Further, among national control programs, we designated the category “low coverage” where snail control, or MDA, or both were included, but the program achieved low (<30%) coverage for either strategy. Similarly, engineering controls were considered present only where their coverage was high (i.e. where more than 30% coverage was achieved).

**Table 1:** Countries and territories evaluated and their success categories, prevalence reduction, and percent reduction in population at risk for schistosomiasis (as a proportion of the total population).

			Prevalence reduction (%)		Population at risk reduction (%)	
Country/territory name	Historical or contemporary national schistosomiasis control program?	Control program outcome (success category)	Baseline: post-control	Baseline: now	Baseline: post-control	Baseline: now
Algeria	ND	Not enough data	ND	ND	ND	ND
Angola	No	Minimal control	NA	ND	NA	ND
Antigua	No	Fortuitous elimination	NA	100	NA	100
Benin	Yes	Not (yet) successful	-2	-2	ND	ND
Botswana	No	Minimal control	NA	NA	NA	NA
Brazil	Yes	Not (yet) successful	92	80	72	69
Burkina Faso	Yes	Not (yet) successful	61.2	61.2	ND	ND
Burundi	Yes	Not (yet) successful	74.4	74.4	ND	ND
Cambodia	Yes	Not (yet) successful	83	83	90	90
Cameroon	Yes	Not (yet) successful	16.7	16.7	ND	ND
Cape Verde	---	Never endemic	NA	NA	NA	NA
Cen. African Republic	Yes	Not (yet) successful	-58	-58	ND	ND
Chad	No	Minimal control	NA	NA	NA	ND
China	Yes	Not (yet) successful	98.9	98.9	79	79
Comoros	---	Never endemic	NA	NA	NA	NA
Congo	Yes	Not (yet) successful	-58	41.7	ND	ND
Côte d'Ivoire	No	Minimal control	NA	NA	NA	NA
Dem. Rep. of the Congo	No	Minimal control	NA	NA	NA	NA
Djibouti	No	Fortuitous elimination	NA	NA	NA	NA
Dominican Republic	Yes	Not (yet) successful	ND	ND	ND	ND
Egypt	Yes	Not (yet) successful	99	99	ND	ND
Equatorial Guinea	No	Minimal control	NA	NA	NA	NA
Eritrea	No	Minimal control	NA	NA	NA	NA
Ethiopia	No*	Minimal control	NA	NA	NA	NA
Gabon	No	Minimal control	NA	NA	NA	NA
Gambia	No	Minimal control	NA	NA	NA	NA
Ghana	Yes	Not (yet) successful	73.9	73.9	ND	ND
Guadeloupe	Yes	Not (yet) successful	38	96	-16	ND
Guinea	No*	Minimal control	NA	NA	NA	NA
Guinea-Bissau	No	Minimal control	NA	NA	NA	NA
Indonesia	Yes	Not (yet) successful	99.5	99.5	90	ND
Iran	Yes	Successful	99.5	100	88.6	100
Iraq	Yes	Not (yet) successful	99.4	99.5	63	ND
Japan	Yes	Successful	100	100	100	100
Jordan	Yes	Successful	100	100	100	100
Kenya	Yes	Not (yet) successful	51	51	ND	ND
Laos	Yes	Not (yet) successful	96	84.6	4.7	ND
Lebanon	Yes	Successful	100	100	100	100
Lesotho	No	Minimal control	NA	NA	NA	NA
Liberia	No*	Minimal control	ND	ND	ND	ND
Libya	Yes	Not (yet) successful	66.7	66.7	NA	NA
Madagascar	Yes	Not (yet) successful	5.4	73.8	-14.6	ND
Malawi	Yes	Not (yet) successful	81.8	43.3	ND	ND
Malaysia	No	Fortuitous elimination	NA	NA	NA	NA
Mali	Yes	Not (yet) successful	50.6	51.8	-88	ND
Martinique	Yes	Successful	88.9	100	100	100
Mauritania	No	Minimal control	NA	NA	NA	NA
Mauritius	Yes	Successful	35.7	100	0	100
Montserrat	No	Fortuitous elimination	NA	NA	NA	NA
Morocco	Yes	Successful	100	100	100	100
Mozambique	Yes	Not (yet) successful	28.9	28.9	ND	ND
Namibia	No*	Minimal control	NA	NA	NA	NA
Niger	Yes	Not (yet) successful	50	44	ND	ND
Nigeria	No	Minimal control	NA	NA	NA	NA
Oman	Yes	Not (yet) successful	3	0.6	-1025	ND
Pakistan	---	Never endemic	NA	NA	NA	NA
Philippines	Yes	Not (yet) successful	93	98.3	11	ND
Puerto Rico	Yes	Successful	85.3	100	78.4	100
Rwanda	Yes	Not (yet) successful	69.5	69.5	ND	ND
Sao Tome & Principe	No	Minimal control	NA	NA	NA	NA
Saudi Arabia	Yes	Not (yet) successful	81.05	99.8	-18	ND
Senegal	Yes	Not (yet) successful	1	1	ND	ND
Seychelles	---	Never endemic	NA	NA	NA	NA
Sierra Leone	Yes	Not (yet) successful	51.4	51.4	ND	ND
Somalia	Yes	Not (yet) successful	-24	-24	NA	NA
South Africa	No	Minimal control	NA	NA	NA	NA
St. Lucia	Yes	Not (yet) successful	88	98.2	84.3	84.3
Sudan	Yes	Not (yet) successful	-29.7	-29.7	47	47
Surinam	Yes	Not (yet) successful	61.5	61.5	69.3	69.3
Swaziland	Yes	Not (yet) successful	9.6	9.6	ND	ND
Syria	Yes	Not (yet) successful	65.4	ND	38	ND
Tanzania	Yes	Not (yet) successful	60	60	0	ND
Thailand	No	Fortuitous elimination	NA	NA	NA	NA
Togo	Yes	Not (yet) successful	30.9	30.9	ND	ND
Tunisia	Yes	Successful	100	100	100	100
Turkey	No	Fortuitous elimination	NA	NA	NA	NA
Uganda	Yes	Not (yet) successful	55.4	55.4	ND	ND
Venezuela	Yes	Not (yet) successful	90	98.6	ND	ND
Western Sahara	ND	not enough data	ND	ND	ND	ND
Yemen	Yes	Not (yet) successful	44	44	ND	ND
Zambia	Yes	Not (yet) successful	26.6	26.6	ND	ND
Zanzibar	Yes	Not (yet) successful	76.6	84.7	ND	ND
Zimbabwe	No*	Minimal control	NA	NA	NA	NA

Negative values represent increases.

“Baseline: post-control” compares just before to just after the control program.

“Baseline: now” refers to just before compared with contemporary estimates.

NA = not applicable.

ND = no data.

*Indicates that a control program has begun, but too recently (2012 or later) to evaluate its nationwide effect: in Ethiopia, control began 2015; in Guinea, control began 2012; in Liberia, control began 2012; in Namibia, control using praziquantel is planned but not yet started in 2015; in Zimbabwe, control began 2012)

To test the hypothesis that control strategies differed in their ability to reduce prevalence, we used a quantitative generalized linear mixed model (GLMM, function “glmer” from the R package “lme4” ([42], S2 Table). The statistical model assessed what factors best predicted relative change in prevalence over time for the 44 countries that applied concerted control and had quantitative, longitudinal data on prevalence, control strategies and covariates (more details below and in S2 Table). This GLMM considered country as a random effect (to account for the repeated measures over time within each country) and the following fixed effects: (i) control program duration (to test if longer efforts might be more successful); (ii) a country’s status as an island or mainland; (iii) initial prevalence before control began (to account for the control effort needed); (iv) the percentage of the population with access to improved water sources (World Bank Indicators, 2012 [43]); and (v) the inflation-adjusted per capita GDP over time (recorded at each time point with disease data from The Maddison-Project [47]). We were most interested in the interaction terms between the predictors and time [year], which, if significant, would indicate an effect on prevalence reduction or increase over time. We first assembled a “full model” that contained all predictors and interaction terms, and then used a model selection procedure based on Akaike’s information criterion (AIC) to remove each interaction term in turn to find the best balance between parsimony and fit to the data ([48], S3 Table).

After analyzing what control strategies were most successful, we became curious about the factors that might have determined which control strategies a country used. To that end, we assessed the correlations between the control strategies used and a country’s “wealth” status (per-capita GDP for each country at each time-point) as well as the control era (the year each national- or territory-level control program began).

## Results

**Fig 4:**
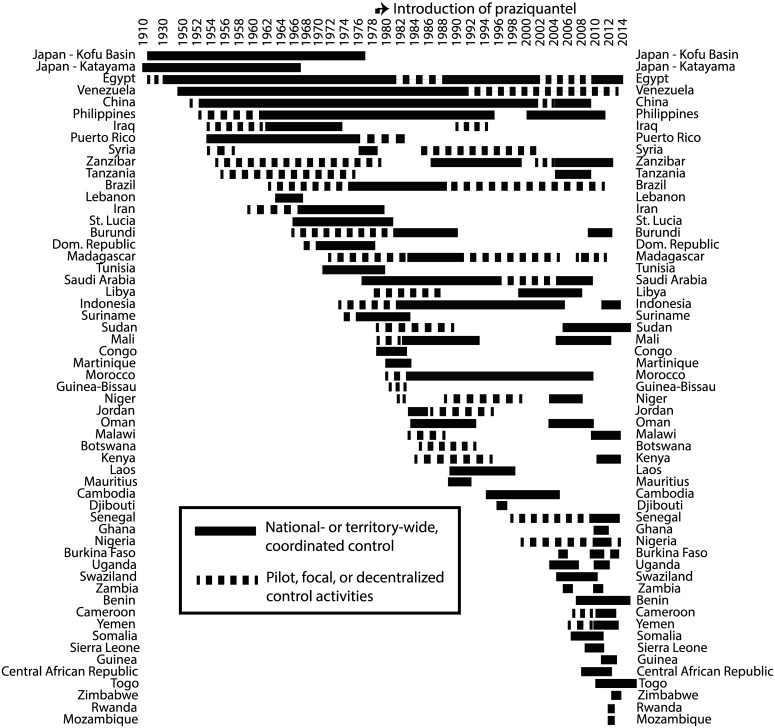
Schistosomiasis control programs over time. See Fig 5 for more details on control strategies and outcomes.

The response to schistosomiasis varied across the 83 evaluated countries/territories. Seventy-two countries and five territories were “endemic” for schistosomiasis during the 20^th^ century (whereas four were probably “never endemic,” and two had too little data to determine their historical or contemporary schistosomiasis endemicity; Table 1, Fig 3). Only 51 (66%) of endemic countries/territories had coordinated national- or territory-level schistosomiasis control during the past century, whereas the remaining 26 (34%) had no verifiable programs (despite some having small pilot programs; Table 1, Figs 3 and 4). Nine endemic countries/territories—Iran, Japan, Jordan, Lebanon, Martinique, Mauritius, Morocco, Puerto Rico, and Tunisia—applied coordinated control programs and achieved “success” as we defined it here.

Nine countries/territories that have not yet achieved schistosomiasis elimination achieved a 90% or greater reduction in their country-level prevalence since baseline (before control): China, Egypt, Guadeloupe, Indonesia, Iraq, Philippines, Saudi Arabia, St. Lucia, and Venezuela. The remaining countries had variable schistosomiasis prevalence over the past century (Table 1), depending, in part, on their control strategy.

### Elimination Predictors

Counter to expectations, elimination/non-endemicity was not associated with having a control program. This unexpected result was due to two factors: several countries/territories achieved “fortuitous” elimination without any documented control effort (Antigua, Djibouti, Malaysia, Montserrat, Thailand, and Turkey) and several other countries failed to eliminate schistosomiasis, despite substantial prevalence reductions. Island/mainland did not predict elimination status (Table 2), however, our inclusion of population size, which is higher on continents and makes elimination harder, could have co-varied with a mainland-island effect. Possessing greater “wealth” (indicated by a higher contemporary per capita GDP) did not affect elimination. Elimination was, however, more likely where more people can access improved water sources. In summary, achieving elimination was idiosyncratic. It was easier with smaller infected populations and in countries with improved (safer) water sources. Although many programs have failed to eliminate schistosomiasis, sometimes elimination has occurred without a coordinated control program. Below, we discuss what factors in addition to control programs could affect schistosomiasis prevalence reductions and elimination success.

**Table 2:** Logistic regression for elimination/non-endemicity. *

Predictor	Estimate	p value
Control program?–presence/absence	1.22	0.42
Population infected before control (log-transformed)	**-0.74**	0.028*
Improved water source (rural % with access), 2012	**0.16**	0.027*
GDP per capita (2013)	-0.00004	0.55
Island? Yes/no	-0.15	0.87

*see S2 Table for more statistical details; N = 68 countries/territories evaluated

### Effective Control Strategies—Quantitative Outcomes

**Fig 5:**
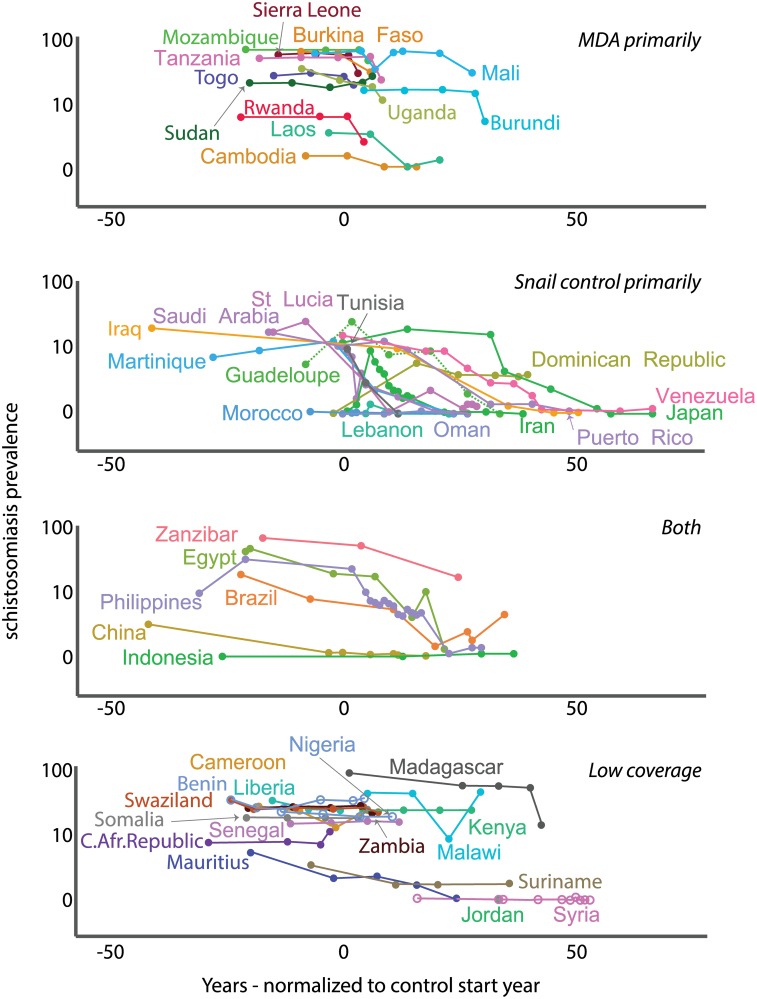
Schistosomiasis prevalence change over time. Prevalence change by control program strategy (time 0 on the x-axis is set when control began; negative values for the normalized year show data *n* years before control started and positive values *n* years after).

**Fig 6:**
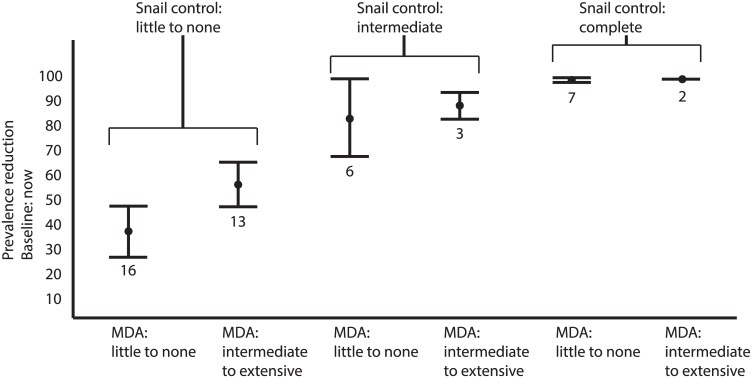
Prevalence change by extent of snail control, or MDA, or both—see text for category definitions.

Although fortuitous elimination in several countries confounded our ability to assess whether control programs eliminated schistosomiasis, many areas with control programs experienced durable *prevalence reductions*. A program’s effectiveness (i.e., the prevalence reduction rate) depended strongly on strategy type and coverage and weakly on the intercept (prevalence at baseline). Applying snail control, MDA, or both—with at least intermediate (>30%) coverage—worked better than any programs with low coverage. Snail control programs (primarily mollusciciding and biological control using non-native, competitor snails) showed the strongest prevalence reductions (while accounting for other covariates, including: control duration [in years], country “wealth” [as per capita GDP in each year with disease data], and access to improved water sources; Table 3, Figs 5 and 6). In other words, all else being equal, prevalence reduction was highest with snail control at intermediate or better coverage.

**Table 3:** Generalized linear mixed model (GLMM, see S2 and S3 Tables) comparing change in prevalence for control programs using: MDA with praziquantel, snail control primarily (snail control), both (MDA + snail control), or minimal control (<30% coverage, “low coverage,” not shown).

Description	Predictor	Estimate* (X 10^−2^)	p-value
Interaction terms**	Control duration:time	**0.02**	**<0.001**
	Island:time	**1.1**	**<0.001**
	Improved water (contemporary % with access):time	**0.09**	**<0.001**
	GDP per capita (inflation adjusted):time	**1.2**	**0.0013**
	Baseline prevalence:time	**-0.04**	**<0.001**
	Engineering controls:time	0.09	0.79
	MDA:time	**-2.3**	**<0.001**
	Snail control:time	**-12.3**	**<0.001**
	Both (MDA + snail control):time	**-7.9**	**<0.001**

*The estimate indicates the change in prevalence over time where more negative (positive) values indicate stronger prevalence reduction (increase) associated with that predictor.

Random effects for country/territory (intercept): variance = 2.06, std. dev. 1.44

N = 44 countries/territories evaluated.

**These are predictors association with change in prevalence over time (years) in each country/territory

Although engineering controls (e.g., installing sanitation infrastructure, cementing canals, building bridges, or draining wetlands), were almost always accompanied by snail control, about half of the programs using snail control did not use engineering in their control programs. Programs that used MDA as a primary strategy (without snail control) also did not report using any large-scale engineering controls. The presence or absence and extent of engineering controls showed weak effects on prevalence, and including 3-way interactions with this variable—along with the other control strategies and time—in the quantitative statistical model did not improve model fit to the data (based on AIC; S3 Table). Thus, engineering controls, although perhaps beneficial within some integrated programs, did not consistently reduce schistosomiasis prevalence.

**Fig 7:**
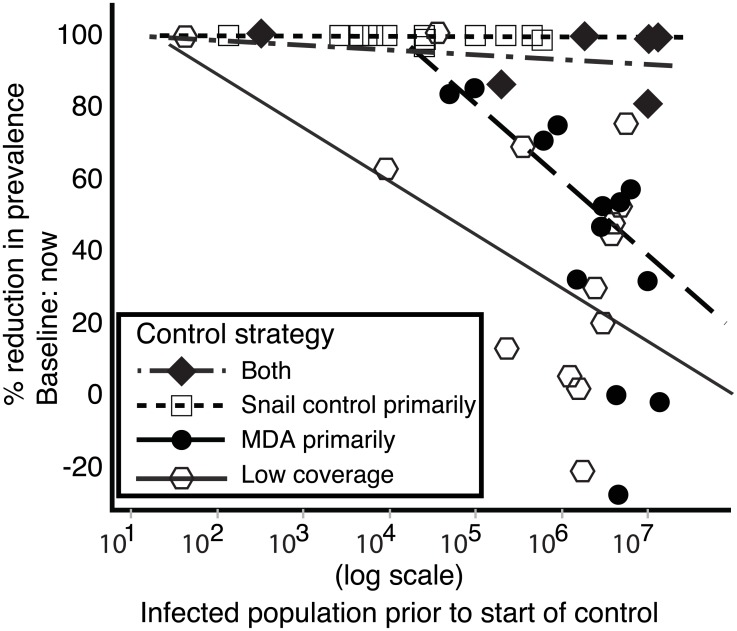
Sensitivity of schistosome prevalence reduction to the infected population size before the control program began.

Population size affected control success. As expected, prevalence reductions were impaired where there were larger initial infected human populations, but this relationship differed among the control strategies. Snail control programs (with or without MDA) were less sensitive to initial infected human population size, than were other approaches (Fig 7).

### Wealth and Era Effects

**Fig 8:**
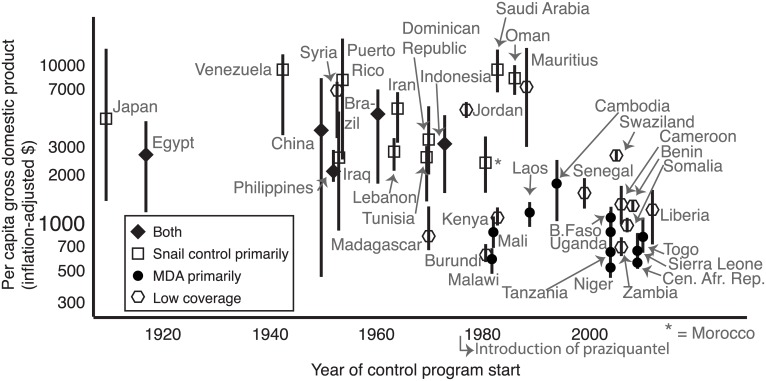
Per capita gross domestic product (GDP) as it relates to control strategy and control start date in each country. Points represent mean inflation-adjusted, per-capita GDP throughout the relevant time for which disease data were available, and bars represent the range.

Control strategy depended on country wealth and the year in which control began. Richer countries (measured by inflation-adjusted, per-capita GDP) tended to begin their control programs earlier in the 20^th^ century, with a stronger focus on snail control and greater success (Fig 8). Higher wealth was also correlated with greater access to improved (safer) water sources.

Large-scale MDA programs were rare before praziquantel entered the global market in the 1980s. After this turning point, there was a new option (using both MDA and snail control) and this integrated strategy has been used since the 1980s in places like China, Egypt, and Brazil. Countries that began their control programs even more recently (after the 1990s or 2000s) were poorer and tended either to focus on MDA or achieved poor coverage (designated as “low coverage” in Fig 8).

## Discussion

Our results support recent suggestions that snail control is key to schistosomiasis reduction [5,6,49]. Such an effect has been anticipated. In 1985, a lead researcher of the Caribbean “St. Lucia Project,”–a Rockefeller-funded schistosomiasis control study—wrote, “chemotherapy is now assuming the major role in control programmes, but in most… a reservoir of infection inevitably remains. Transmission is thus likely to continue at a low, but probably increasing level unless a supplementary control strategy is present” [24]. Unfortunately, it took decades to assess this prediction.

Chemotherapy has major benefits for infected humans, but, by itself, MDA has done little to curb re-infection. Although programs limited to MDA with praziquantel did not appear to do as well as the other strategies evaluated, the (targeted) chemotherapy for infection control remains an undeniable factor in improving health, especially when integrated with snail control. Countries whose programs focused on snail control often relied on distributing chemotherapy through means other than MDA, such as Morocco’s successful test-and-treat (TAT) campaigns using mobile teams [50], Iraq’s early school-based TAT programs [51], and Japan’s involvement as an early TAT site for praziquantel beginning in the late 1970s. This involvement might have carried Japan to country-wide elimination by 1996 [52]. One reason praziquantel seems less effective than expected is that it was applied later in history when control campaigns targeted more challenging countries. In other words, schistosomiasis elimination was more successful among programs started before praziquantel reached the global market than among those programs started after the drug’s introduction in the late 1970s. This might arise, in part, because wealthier countries tended to address the disease earlier in the 20^th^ century, as they could afford molluscicides for widespread snail control (although inexpensive biological control also sometimes succeeded).

The “fortuitous elimination” of schistosomiasis from Antigua, Djibouti, Malaysia, Montserrat, Thailand, and Turkey without documented control efforts suggests cryptic factors have affected schistosomiasis, including: species invasions (e.g. snail competitors or predators), sanitation or health care improvements outside control programs, and human-induced or natural ecosystem changes (such as changes in dams, irrigated-agriculture, and urbanization). The least fortuitous of the fortuitous eliminations was the 1995 volcanic eruption that drove almost half of Montserrat’s population off the island and made the schistosomiasis transmission zones off limits to people [53,54]. (See S2 Appendix and S1 Fig for more cryptic schistosomiasis control examples).

These results suggest that programs have been most effective when snail control is coordinated soon thereafter—or simultaneously—with chemotherapy (morbidity control) via a rational progression from widespread, active drug distribution campaigns (MDA or targeted treatment (TAT)) to a focus on high-risk groups and finally passive distribution within health services coupled with surveillance (e.g. “surveillance and response”[55,56]) and health education (e.g. IEC) in the “end game” (Fig 2b).

As for how to control snails, the most common strategy has been to use expensive and toxic molluscicides; an effort that is neither feasible nor desirable for many poor countries where schistosomiasis is now endemic. Schistosomiasis has been hard to control without well-funded, national-level efforts, and the contemporary global health discussion has been focused on strategies that optimize efficiency and affordability. By recognizing the successful use of snail control for transmission reduction, and by fostering research directed toward the development of creative, safe and cheap tools to target the snail intermediate host, global schistosomiasis elimination might be attainable.

## Supporting Information

S1 Appendix Additional references. (DOCX)

S2 Appendix Supporting text. Detailed examples of cryptic social, ecological, and political factors: species invasions, sanitation, and ecosystem change.
(DOCX)

S1 Table List of variables searched for and recorded (when available) to assemble the database analyzed in this paper. (DOCX)

S2 Table Variables included (and regression formulas used) in the binary and quantitative analyses. (DOCX)

S3 Table Model selection results leading to the final model discussed in the main text. “3way interactions” signify a Strategy*Year*Engineering term in the full-model. The other variables are as in S2 Table. Those models above the dotted line were deemed to fit the data best. Removing the 3way interactions with engineering controls did not substantially change the model fit to data, whereas removing the other variables decreased the fit (increased Akaike’s information criterion (AIC)) substantially.
(DOCX)

S1 Fig Progression of schistosomiasis prevalence reductions (black lines) and timing of alien species introductions (intentional or inadvertent) which led to biological invasions of competitor snails (solid arrows) in the Caribbean or crayfish (dashed arrow) in Egypt. The suspected time-course of biological invasions in these regions—with most invasions occurring just before or during periods of maximal prevalence reduction—suggests the plausibility that invasions may have influenced schistosomiasis control outcomes for many, if not all, of these case studies.
(DOCX)
