# Supplementary material for: Global Assessment of Schistosomiasis Control Over the Past Century Shows Targeting the Snail Intermediate Host Works Best
Source: PLoS Negl Trop Dis. 2016 Jul 21;10(7):e0004794. doi: 10.1371/journal.pntd.0004794 (PMC4956325; doi:10.1371/journal.pntd.0004794)
Supplement: S1 Appendix — (DOCX) [file pntd.0004794.s001.docx]

**S1 Appendix: Additional references list**

Abdel-Azim M, Gismann A (1956) Bilharziasis survey in south-western Asia; covering Iraq, Israel, Jordan, Lebanon, Sa'udi Arabia, and Syria: 1950-51. Bulletin of the World Health Organization 14: 403-456.

Agbo K, Sodahlon YK, Clocuh F, Dogba M (1999) Prevalence des schistosomoses au Togo etude transversale realisee en milieu scolaire. Medecine Tropicale 59: 51-54.

Al-Juburi AZ, Mukhlis G, Muhsen J, Miller HC (1987) Schistosomiasis: a twenty-year review. The Journal of the Arkansas Medical Society 84: 47-51.

Al-Madani AA (1990) Schistosomiasis control in Saudi Arabia with special reference to the period 1983-1988. Public Health 104: 261-266.

Alberda A, Weits J, Limburg AJ, Grond J, Ilic P (1987) Chronic schistosomiasis in Surinam subjects: symptoms, treatment and course. Nederlands tijdschrift voor geneeskunde 131: 2308-2312.

Albonico M, Shamlaye N (1996) Control of intestinal parasitic infections in Seychelles: a comprehensive and sustainable approach. Bulletin of the World Bank 74: 577-586.

Almeda J, Corachan M, Sousa A, Ascaso C, Carvalho JM, et al. (1994) Schistosomiasis in the Republic of São Tomé and Principe: human studies. Transactions of the Royal Society of Tropical Medicine and Hygiene 88: 406-409.

Almeida Machado P (1982) The Brazilian program for schistosomiasis control, 1975-1979. American Journal of Tropical Medicine and Hygiene 31: 76-86.

Amaral RSd, Tauil PL, Lima DD, Engels D (2006) An analysis of the impact of the Schistosomiasis Control Programme in Brazil. Memórias do Instituto Oswaldo Cruz. pp. 79-85.

Amarir F, El Mansouri B, Fellah H, Sebti F, Mohammed L, et al. (2011) National serologic survey of Haematobium schistosomiasis in Morocco: Evidence for elimination. American Journal of Tropical Medicine and Hygiene 84: 15-19.

Amarir F, Sebti F, Abbasi I, Sadak A, Fellah H, et al. (2014) *Schistosoma haematobium* detection in snails by DraI PCR and Sh110/Sm-Sl PCR: further evidence of the interruption of schistosomiasis transmission in Morocco. Parasites & vectors 7: 288.

Anon. Schistosomiasis Control Initiative: Mozambique strategy. [cited 2015 Dec 30]; Available from: <http://www3.imperial.ac.uk/schisto/wherewework/mozambique/mozambiquestrategy>.

Anon. Schistosomiasis Control Initiative: Rwandan strategy. [cited 2015 Dec 30]; Available from: <http://www3.imperial.ac.uk/schisto/wherewework/rwanda/rwandastrategy>.

Anon. World Bank: World Development Indicators. [cited 2015; databank]. Available from: <http://databank.worldbank.org/data/reports.aspx?source=world-development-indicators>.

Anon. (1919) Bilharziasis : its Prevention and Treatment. The Lancet 193: 1032.

Anon. (1999) Regional Disease Vector Ecology Profile: The Middle East: Defense Pest Management Information Analysis Center, Armed Forces Pest Management Board, Forest Glen Section, Walter Reed Army Medical Center.

Anon. (2005) Etat d’avancement des programmes de lutte contre les maladies parasitaires. Direction de l’Épidémiologie et de Lutte contre les Maladies, Division des Maladies transmis- sibles, Service des Maladies parasitaires. 39-51 p.

Anon. (2010) Republic of Yemen - Schistosomiasis Project. Washington, D.C.: World Bank. AB4144 AB4144.

Anon. (2013) Operational Plan 2013 for Neglected Tropical Disease Control in Burkina Faso Annual Work Plan. New York: Hellen Keller International. 1-26 p.

Anon. The World Bank Yemen Overview. 2014 [cited 2015 June 15 2015]; Available from: [http://www.worldbank.org/en/country/yemen/overview - 1](http://www.worldbank.org/en/country/yemen/overview#1).

Anon. Fighting disease: Nigeria: Targeting schistosomiasis. 2015 [cited 2015 Dec 31]; Available from: <http://www.cartercenter.org/countries/nigeria-health-schistosomiasis.html>.

Anon. Mass Drug Administration Launch. 2015 [cited 2015 Dec 31]; Ministry of Health and Child Care]. Available from: <http://www.mohcc.gov.zw/request-for-quotation/9-uncategorised/321-mass-drug-administration-launch?showall=1&limitstart=>.

Anon. World Schistosomiasis Risk Chart: Geographical distribution of schistosomiasis and principal snail vectors. 2015 [cited 2015 Dec 31]; 2015 Edition:[1-5]. Available from: https://<http://www.iamat.org/risks/schistosomiasis>.

Anwar AH, Gill SA (1990) A study on ecology of schistosomiasis in cattle and buffaloes. 10: 86-87.

Appleton CC, Ellery WN, Byskov J, Mogkweetsinyana SS (2008) Epidemic transmission of intestinal schistosomiasis in the seasonal part of the Okavango Delta, Botswana. Ann Trop Med Parasitol 102: 611-623.

Arbaji A, Amr ZS, Abbas AA, al-Oran R, al-Kharabsheh S, et al. (1998) New sites of *Bulinus truncatus* and indigenous cases of urinary schistosomiasis in Jordan. Parasite (Paris, France) 5: 379-382.

Arfaa F (1975) Studies on schistosomiasis in Somalia. American Journal of Tropical Medicine and Hygiene 24: 280-283.

Arfaa F, Bijan H, Farahmandian I (1967) Present status of urinary bilharziasis in Iran. Transactions of the Royal Society of Tropical Medicine and Hygiene 61: 358-367.

Arfaa F, Farahmandian I (1970) Progress towards the control of bilharziasis in Iran. Transactions of the Royal Society of Tropical Medicine and Hygiene 64: 912-917.

Arshad G, Maqbool A, Qamar M, Muhammad S, Bukhari H (2011) Prevalence and ecology of freshwater snails in some selected districts of Southern Punjab, Pakistan. Pakistan Journal of Life and Social Sciences 9: 17-20.

Arshad GM, Maqbool A, Qamar MF, Bukhari SMH, Hashmi HA, et al. (2011) Epidemiology of schistosomiasis in buffaloes under different managemental conditions in four districts of Punjab, Pakistan. Journal of Animal and Plant Sciences 21: 841-843.

Attwood SW (2001) Schistosomiasis in the Mekong region: Epidemiology and phylogeography. Advances in Parasitology. pp. 87-152.

Attwood SW (2005) *Robertsiella silvicola*, a new species of triculine snail (Caenogastropoda: Pomatiopsidae) from Peninsular Malaysia, intermediate host of *Schistosoma malayensis* (Trematoda: Digenea). Journal of Molluscan Studies 71: 379-391.

Attwood SW, Upatham E (2012) Observations on *Neotricula aperta* (Gastropoda: Pomatiopsidae) population densities in Thailand and central Laos: implications for the spread of Mekong schistosomiasis. Parasites & Vectors. pp. 126.

Augusto G, Nala R, Casmo V, Sabonete A, Mapaco L, et al. (2009) Geographic distribution and prevalence of schistosomiasis and soil-transmitted helminths among schoolchildren in Mozambique. Am J Trop Med Hyg 81: 799-803.

Ayad N (1956) Bilharziasis survey in British Somaliland, Eritrea, Ethiopia, Somalia, the Sudan, and Yemen. Bulletin of the World Health Organization 14: 1-117.

Azar JE, Luttermoser G, Schacher JF (1961) First report of a focus of schistosomiasis in Lebanon. American Journal of Tropical Medicine and Hygiene 10: 709-711.

Baquir H (1974) Letter: Present status of Hor Rajab bilharziasis control project Iraq 15, WHO-TA. Transactions of the Royal Society of Tropical Medicine and Hygiene 68: 345.

Barakat R, El Morshedy H (2011) Efficacy of two praziquantel treatments among primary school children in an area of high Schistosoma mansoni endemicity, Nile Delta, Egypt. Parasitology 138: 440-446.

Barakat R, Morshedy HE, Farghaly A (2014) Human Schistosomiasis in the Middle East and North Africa Region. Vienna: Springer Vienna. 978-3-7091-1612-8 978-3-7091-1612-8.

Barakat RMRR (2013) Epidemiology of schistosomiasis in Egypt: Travel through time: Review. Cairo University Journal of Advanced Research 4: 425-432.

Barbosa FS, Pinto R, Souza OA (1971) Control of schistosomiasis mansoni in a small Northeast Brazilian community. Transactions of the Royal Society of Tropical Mediciine Hygiene 65: 206-213.

Barbosa FS, Sanches O, Barbosa CS, Arruda F (1992) Dynamics of snail populations of *Biomphalaria glabrata* and *B. straminea* under semi-natural conditions. Cadernos de Saúde Pública.

Barkia H, Barkia A, Belghyti HND (2011) La schistosomiase au Maroc : de sa découverte à l ’ après-élimination. Eastern Mediterranean Health Journal 17: 250-256.

Barkia H, Barkia A, Yacoubi R, Alemad A, Kharim EL, et al. (2014) Contribution of mobile teams to efforts to eliminate schistosomiasis at *Schistosoma haematobium* in Morocco: Narrative Review Article. Iranian Journal of Public Health 43: 1167-1175.

Baruffa G, Friedheim Ea (1960) Antimony dimercaptosuccinate (TWSb) in the treatment of urinary bilharziasis in Somalia. The Central African Journal of Medicine 6: 485-487.

Berger S (1995) Infectious diseases of Namibia. Los Angeles: Gideon Informatics, Inc. pp. 400.

Bergquist R, Tanner M (2010) Controlling schistosomiasis in Southeast Asia: a tale of two countries. Advances in parasitology 72: 109-144.

Blacklock DB (1925) Endemic goitre and schistosomiasis in Sierra Leone. Transactions of the Royal Society of Tropical Medicine and Hygiene 18: 395-416.

Blacklock DB (1925) Endemic goitre and schistosomiasis in Sierra Leone: Part II: Schistosomiasis in Sierra Leone. Transactions of the Royal Society of Tropical Medicine and Hygiene 18: 395-416.

Blas BL, Rosales MI, Lipayon IL, Yasuraoka K, Matsuda H, et al. (2004) The schistosomiasis problem in the Philippines: a review. Parasitology international 53: 127-134.

Boelee E, Laamrani H (2004) Environmental control of schistosomiasis through community participation in a Moroccan oasis. Tropical Medicine and International Health 9: 997-1004.

Borrmann S, Szlezák N, Faucher JF, Matsiegui PB, Neubauer R, et al. (2001) Artesunate and praziquantel for the treatment of *Schistosoma haematobium* infections: a double-blind, randomized, placebo-controlled study. The Journal of infectious diseases 184: 1363-1366.

Botelho MC, Machado A, Carvalho A, Valca M, Conceicao O, et al. (2015) Urinary schistosomiasis in Guinea-Bissau. Tropical Medicine and International Health 20: 239.

Bowie C, Purcell B, Shaba B, Makaula P, Perez M (2004) A national survey of the prevalence of schistosomiasis and soil transmitted helminths in Malawi. BMC infectious diseases 4: 49.

Brooker S, Rowlands M, Haller L, Savioli L, Bundy Da (2000) Towards an atlas of human helminth infection in sub-Saharan Africa: the use of geographical information systems (GIS). Parasitology today (Personal ed) 16: 303-307.

Brown DS (1991) Freshwater snails of Sao Tome, with special reference to *Bulinus forskalii* (Ehrenberg), host of *Schistosoma intercalatum*. Hydrobiologia 209: 141-153.

Bundy DAP, Wong MS, Lewis LL, Horton J (1990) Control of geohelminths schools by delivery of targeted chemotherapy through schools. Transactions of the Royal Society of Tropical Medicine Hygiene 84: 115-120.

Catto J (1904) A new trematode. British Medical Journal 2: 663-664.

Cawston FG (1918) Bilharziasis in South Africa. The Journal of American Medical Association 70: 439-441.

Cawston FG (1921) *Schistosoma mansoni* in South Africa. The Lancet 198: 332.

Chapotin C (1812) Maladies de voies urinaires. Paris: Didot Jeunne.

Chevreau P, Chazal EL (1890) Etude sur le bilarzia hematobia à l'Ile Maurice. Bulletin de la Société Médicale de l'Ile Maurice.

Chimbari MJ (2012) Enhancing schistosomiasis control strategy for Zimbabwe: building on past experiences. Journal of parasitology research 2012: 1-9.

Chimbari MJ, Dhlomo E, Mwadiwa E, Mubila L (2003) Transmission of schistosomiasis in Kariba, Zimbabwe, and a cross-sectional comparison of schistosomiasis prevalences and intensities in the town with those in Siavonga in Zambia. Ann Trop Med Parasitol 97: 605-616.

Chitsulo L, Engels D, Montresor A, Savioli L (2000) The global status of schistosomiasis and its control. Acta tropica 77: 41-51.

Christopherson, J.B. (1918) The use of antimony in bilharziosis. The Lancet: 325-327.

Chu KY, Massoud J, Arfaa F (1968) Distribution and ecology of *Bulinus truncatus* in Khuzestan, Iran. Bulletin of the World Health Organization 39: 607-637.

Chu TB, Liao CW, Huang YC, Chang YT, Costa aSRJ, et al. (2012) Prevalence of *Schistosoma intercalatum* and *S. haematobium* infection among primary schoolchildren in capital areas of democratic republic of São Tomé and Príncipe, West Africa. Iranian Journal of Parasitology 7: 67-72.

Clements ACA, Brooker S, Nyandindi U, Fenwick A, Blair L (2008) Bayesian spatial analysis of a national urinary schistosomiasis questionnaire to assist geographic targeting of schistosomiasis control in Tanzania, East Africa. International journal for parasitology 38: 401-415.

Clements ACA, Garba A, Sacko M, Touré S, Dembelé R, et al. (2008) Mapping the probability of schistosomiasis and associated uncertainty, West Africa. Emerging infectious diseases 14: 1629-1632.

Clemow FG (1903) The Geography of Disease. London: C.J. Clay and Sons, Cambridge University Press.

Colston J, Saboyá M (2013) Soil-transmitted helminthiasis in Latin America and the Caribbean: Modelling the determinants, prevalence, population at risk and costs of control at sub-national level. Geospatial Health 7: 321-340.

Corachan M, Romero R, Mas J, Palacin A, Knowles R (1988) A case of *Schistosoma intercalatum* infection from Sao Tome. Tropical and geographical medicine 40: 147-150.

Coura JR, Amaral RS (2004) Epidemiological and control aspects of schistosomiasis in Brazilian endemic areas. Memorias do Instituto Oswaldo Cruz. pp. 13-19.

Cowper SG (1953) Schistosomiasis in Mauritius. Transactions of the Royal Society of Tropical Medicine and Hygiene 47: 564-579.

Dabo A, Doucoure B, Koita O, Diallo M, Kouriba B, et al. (2000) Reinfection with *Schistosoma haematobium* and *mansoni* despite repeated praziquantel office treatment in Niger, Mali. MedTrop(Mars) 60: 351-355.

de Clercq D, Rollinson, D., Diarra, A., Sacko, M., Coulibaly, G., Landoure ́, A., et al. (1994) Schistosomiasis in Dogon country, Mali: identification and prevalence of the species responsible for infection in the local community. Royal Society of Tropical Medicine and Hygiene 88: 653-665.

de Jonge N, van Vliet NG (1991) Urinary schistosomiasis in the Sichili Health Zone, Western Province, Zambia. Tropical Medicine and Parasitology 42: 191-192.

De Noya BA, Balzan C, Arteaga C, Cesari I, Noya O (1999) The last fifteen years of schistosomiasis in Venezuela: Features and evolution. Memorias do Instituto Oswaldo Cruz 94: 139-146.

de Noya BA, Noya O, Balzan C, Cesari IM (1992) New approaches for the control and eradication of schistosomiasis in Venezuela. Memorias do Instituto Oswaldo Cruz 87: 227-231.

Deribe K, Meribo K, Gebre T, Hailu A, Ali A, et al. (2012) The burden of neglected tropical diseases in Ethiopia, and opportunities for integrated control and elimination. Parasites & Vectors 5: 240.

Dhunputh J (1994) Progress in the control of schistosomiasis in Mauritius. Transactions of the Royal Society of Tropical Medicine and Hygiene 88: 507-509.

Doumenge J, Mott K, Cheung C, Villenave D, Chapuis O, et al. Atlas of the global distribution of schistosomiasis. 1987; Available from: <http://www.who.int/schistosomiasis/epidemiology/global_atlas/en/>.

Ebanks GE (1988) The aging of the population of Montserrat : Causes and consequences. Caribbean Studies 21: 101-122.

El Khoby T, Galal N, Fenwick a (1998) The USAID/Government of Egypt's Schistosomiasis Research Project (SRP). Parasitology Today 14: 92-96.

El-Halawani A (1978) Evaluation of molluscicidal control of schistosomiasis in the Middle East. In: Abdallah A, editor. Proceedings of the International Conference on Schistosomiasis. Cairo, Egypt: Egypt Ministry of Health, 1978. pp. 349-357.

El-Khoby T, Galal N, Fenwick A, Barakat R, El-Hawey A, et al. (2000) The epidemiology of schistosomiasis in Egypt: Summary findings in nine governorates. American Journal of Tropical Medicine and Hygiene 62: 88-99.

Engels D, Ndoricimpa J, Gryseels B (1993) Schistosomiasis mansoni in Burundi: Progress in its control since 1985. Bulletin of the World Health Organization 71: 207-214.

Erko B, Gebre-Michael T, Balcha F, Gundersen SG (2001) Implication of *Papio anubis* in the transmission of intestinal schistosomiasis in three new foci in Kime area, Ethiopia. Parasitology International 50: 259-266.

Ernould JC, Kaman aK, Labbo R, Couret D, Chippaux JP (2000) Recent urban growth and urinary schistosomiasis in Niamey, Niger. Tropical Medicine and International Health 5: 431-437.

Facon B, Pointier JP, Glaubrecht M, Poux C, Jarne P, et al. (2003) A molecular phylogeography approach to biological invasions of the New World by parthenogenetic Thiarid snails. Molecular Ecology 12: 3027-3039.

Farooq M, Hairston NG, Samaan SA (1966) The effect of area-wide snail control on the endemicity of bilharziasis in Egypt. Bulletin of the World Health Organization 35: 369-375.

Fenwick A (2011) The control of schistosomiasis in Africa and the evaluation of integrated control of neglected tropical diseases in Africa. Global Health Final Reports Grant ID# 13122 and 36202: Bill & Melinda Gates Foundation.

Fenwick A, Rollinson D, Southgate V (2006) Implementation of human schistosomiasis control: Challenges and prospects. Advances in parasitology 61: 567-622.

Fenwick A, Webster JP, Bosque-Oliva E, Blair L, Fleming FM, et al. (2009) The Schistosomiasis Control Initiative (SCI): rationale, development and implementation from 2002-2008. Parasitology 136: 1719-1730.

Finn TP, Stewart BT, Reid HL, Petty N, Sabasio A, et al. (2012) Integrated rapid mapping of neglected tropical diseases in three States of South Sudan: survey findings and treatment needs. PLoS One 7: e52789.

Fürst T, Silué KD, Ouattara M, N'Goran DN, Adiossan LG, et al. (2012) Schistosomiasis, soil-transmitted helminthiasis, and sociodemographic factors influence quality of life of adults in Côte d'Ivoire. PLoS Neglected Tropical Diseases 6.

Garba A, Barkiré N, Djibo A, Lamine MS, Sofo B, et al. (2010) Schistosomiasis in infants and preschool-aged children: Infection in a single *Schistosoma haematobium* and a mixed *S. haematobium-S. mansoni* foci of Niger. Acta Tropica 115: 212-219.

Garba A, Labbo R, Tohon Z, Sidiki A, Djibrilla A (2004) Emergence of *Schistosoma mansoni* in the Niger River valley, Niger. Transactions of the Royal Society of Tropical Medicine and Hygiene 98: 296-298.

Garba a, Touré S, Dembelé R, Boisier P, Tohon Z, et al. (2009) Present and future schistosomiasis control activities with support from the Schistosomiasis Control Initiative in West Africa. Parasitology 136: 1731-1737.

Garba A, Touré S, Dembelé R, Bosque-Oliva E, Fenwick A (2006) Implementation of national schistosomiasis control programmes in West Africa. Trends in Parasitology 22: 322-326.

Gaud J (1955) Les bilharzioses en Afrique Occidentale et en Afrique Centrale. Bulletin of the World Health Organization 13: 209-258.

Gbakima aa, Moriba MM, Samoh Ma, White PT, Samba Ja (1987) A survey of the prevalence of schistosomiasis in school children in the Bo and Tongo Field areas of Sierra Leone. Public health 101: 199-205.

Geldenhuys PJ, al e (1967) Bilharzia survey in the eastern Caprivi, northern Bechuanaland and northern South West Africa. South African medical journal 41: 767.

Giboda M, Malek EA, Correa R (1997) Human schistosomiasis in Puerto Rico: Reduced prevalence rate and absence of Biomphalaria glabrata. American Journal of Tropical Medicine and Hygiene 57: 564-568.

Gómez Pérez J, Vargas M, Malek EA (1991) Displacement of *Biomphalaria glabrata* by *Thiara granifera* under natural conditions in the Dominican Republic. Memorias do Instituto Oswaldo Cruz 86: 341-347.

Greer GJ, Ambu S, Davis GM (1984) Studies on the habitat, distribution and schistosome infection of *Robertsiella* spp., snail hosts for a *Schistosoma japonicum*-like schistosome in Peninsular Malaysia. Tropical Biomedicine 1: 85-93.

Greer GJ, Dennis DT, Lai PF, Anuar H (1989) Malaysian schistosomiasis: description of a population at risk. The Journal Of Tropical Medicine And Hygiene 92: 203-208.

Greer GJ, Ow-Yang CK, Yong H-S (1988) *Schistosoma malayensis* n. sp.: A *Schistosoma japonicum*-complex schistosome from Peninsular Malaysia. The Journal of Parasitology 74: 471-480.

Gryseels B (1991) The epidemiology of schistosomiasis in Burundi and its consequences for control. Trans R Soc Trop Med Hyg 85: 626-633.

Güralp N (1960) Present situation of schistosomiasis in Turkey. Acta tropica 17: 261-263.

Gyorkos TW, Camara B, Kokoskin É, Carabin H, Prouty R (1995) Enquête de prévalence parasitaire chez les enfants d'âge scolaire en Guinée. Cahiers Santé 6: 377-381.

Haddock KC (1981) Control of schistosomiasis: The Puerto Rican experience. Social Science & Medicine Part D: Medical Geography 15: 501-514.

Haile S, Golassa L, Mekonnen Z (2012) Prevalence of *Schistosoma mansoni* and effectiveness of praziquantel in school children in Finchaa valley, Ethiopia. Journal of Parasitology and Vector Biology 4: 25-30.

Harinasuta C, Kruatrachue M (1962) The first recognized endemic area of bilharziasis in Thailand. Annals of Tropical Medicine and Parasitology 56: 314.

Harmanctoglu N, Alpaslan N, Boelee E (2001) Irrigation, health, and environment: A review of literature from Turkey. Colombo, Sri Lanka.

Harry HW, Aldrich DV (1958) The ecology of *Australorbis glabratus* in Puerto Rico. Bulletin of the World Health Organization 18: 819-832.

Hira PR (1975) Seasonal population densities of snails transmitting urinary and intestinal schistosomiasis in Lusaka, Zambia. Trop Geogr Med 27: 83-92.

Hodges M, Dada N, Wamsley A, Paye J, Nyorkor E, et al. (2011) Improved mapping strategy to better inform policy on the control of schistosomiasis and soil-transmitted helminthiasis in Sierra Leone. Parasites & vectors 4: 97.

Hodges M, Koroma MM, Baldé MS, Turay H, Fofanah I, et al. (2011) Current status of schistosomiasis and soil-transmitted helminthiasis in Beyla and Macenta Prefectures, Forest Guinea. Transactions of the Royal Society of Tropical Medicine and Hygiene 105: 672-674.

Hopkins DR, Eigege A, Miri ES, Gontor I, Ogah G, et al. (2002) Lymphatic filariasis elimination and schistosomiasis control in combination with onchocerciasis control in Nigeria. American Journal of Tropical Medicine and Hygiene 67: 266-272.

Hotez PJ, Bottazzi ME, Franco-Paredes C, Ault SK, Periago MR (2008) The neglected tropical diseases of Latin America and the Caribbean: A review of disease burden and distribution and a roadmap for control and elimination. PLoS Neglected Tropical Diseases.

Hotez PJ, Molyneux DH, Fenwick A, Kumaresan J, Sachs SE, et al. (2007) Control of Neglected Tropical Diseases. The New England Journal of Medicine 357: 1018-1027.

Hotez PJ, Savioli L, Fenwick A (2012) Neglected tropical diseases of the Middle East and North Africa: Review of their prevalence, distribution, and opportunities for control. Plos Neglected Tropical Diseases 6: e1475.

Hotez PJ, Savioli L, Fenwick A (2012) Neglected tropical diseases of the Middle East and North Africa: review of their prevalence, distribution, and opportunities for control. PLoS neglected tropical diseases 6: e1475.

Humaida S, el Gaddal AA, Homeida MA (2011) Schistosomiasis: Epidemiology and burden of disease in the Sudan. Sudan Medical Journal 47: 63-68.

Hunter J, Rey L, Scott D (1982) Man-made lakes and man-made diseases. Social Science and Medicine 16: 1127-1145.

Huyse T, Van den Broeck F, Jombart T, Webster BL, Diaw O, et al. (2013) Regular treatments of praziquantel do not impact on the genetic make-up of *Schistosoma mansoni* in Northern Senegal. Infection, Genetics and Evolution 18: 100-105.

Iarotski LS, Davis A (1981) The schistosomiasis problem in the world: Results of a WHO questionnaire survey. Bulletin of the World Health Organization 59: 115-127.

Idris Ma, Shaban M, Richter J, Moné H, Mouahid G, et al. (2003) Emergence of infections with *Schistosoma manson*i in the Dhofar Governorate, Oman. Acta Tropica 88: 137-144.

Incani RN (1987) The Venezuelan experience in the control of schistosomiasis mansoni. Memorias do Instituto Oswaldo Cruz 82 Suppl 4: 89-93.

Izhar A, Sinaga RM, Sudomo M, Wardiyo ND (2002) Recent situation of schistosomiasis in Indonesia. Acta Tropica. pp. 283-288.

Jember TH (2014) Challenges of schistosomiasis prevention and control in Ethiopia: Literature review and current status. Journal of Parasitology and Vector Biology 6: 80-86.

Jobin WR (1979) Cost of snail control. American Journal of Tropical Medicine and Hygiene 28: 142-154.

Jobin WR, Ferguson FF, Palmer JR (1970) Control of schistosomiasis in Guayama and Arroyo, Puerto Rico. Bulletin of the World Health Organization 42: 151-156.

Jordan P (1985) Schistosomiasis: The St Lucia Project. Cambridge: Cambridge University Press.

Jordan P (2000) From Katayama to the Dakhla Oasis: The beginning of epidemiology and control of bilharzia. Acta Tropica 77: 9-40.

Kabatereine NB, Brooker S, Tukahebwa EM, Kazibwe F, Onapa AW (2004) Epidemiology and geography of *Schistosoma mansoni* in Uganda: Implications for planning control. Tropical Medicine and International Health 9: 372-380.

Kabatereine NB, Fleming FM, Nyandindi U, Mwanza JCL, Blair L (2006) The control of schistosomiasis and soil-transmitted helminths in East Africa. Trends in parasitology 22: 332-339.

Katsivo MN, Muthami LN, Kimani S, Karama M, Kingori F (1993) Involvement of a community in schistosomiasis control: a Kenyan experience. East African Medical Journal. pp. 478-481.

Keiser J, De Castro MC, Maltese MF, Bos R, Tanner M, et al. (2005) Effect of irrigation and large dams on the burden of malaria on a global and regional scale. American Journal of Tropical Medicine and Hygiene 72: 392-406.

Keittivuti B, Keittivuti A, O'Rourke TF (1983) Parasitic diseases with emphasis on schistosomiasis in Cambodian refugees, in Prachinburi Province Thailand. The Southeast Asian Journal of Tropical Medicine and Public Health 14: 491-494.

Khalid SE, Mahmood SM (2001) Schistosomiasis - a viable differential for haematuria in travelers in Pakistan. JPMA The Journal of the Pakistan Medical Association 51: 325-327.

Khalil M, Sleem SH (2011) Can the freshwater crayfish eradicate schistosomiasis in Egypt and Africa? Journal of American Science 7.

King CH (2010) Parasites and poverty: The case of schistosomiasis. Acta Tropica 113: 95-104.

Kloetzel K (1992) Some personal views on the control of schistosomiasis mansoni. Memorias do Instituto Oswaldo Cruz 87 Suppl 4: 221-226.

Kloos H, David R (2002) The paleoepidemiology of schistosomiasis in ancient Egypt. Human Ecology Review. pp. 14-25.

Knopp S, Mohammed Ka, Ali SM, Khamis IS, Ame SM, et al. (2012) Study and implementation of urogenital schistosomiasis elimination in Zanzibar (Unguja and Pemba islands) using an integrated multidisciplinary approach. BMC public health 12: 930.

Knopp S, Person B, Ame SM, Mohammed Ka, Ali SM, et al. (2013) Elimination of schistosomiasis transmission in Zanzibar: baseline findings before the onset of a randomized intervention trial. PLoS neglected tropical diseases 7: e2474.

Knopp S, Stothard JR, Rollinson D, Mohammed Ka, Khamis IS, et al. (2013) From morbidity control to transmission control: time to change tactics against helminths on Unguja Island, Zanzibar. Acta tropica 128: 412-422.

Koeck JL, Modica C, Tual F, Czarnecki E, Fabre R, et al. (1999) Discovery of a focus of intestinal bilharziasis in the Republic of Djibouti. Medecine tropicale: Revue du Corps de Sante Colonial 59: 35-38.

Kruatrachue M (1968) Schistosomiasis in Thailand: Studies on the incidence, epidemiology, and its causing cercarial dermatitis: Final progress report, FE-350-4. San Francisco: U.S. Army Research and Development Group, Defense Technical Information Center. 33 p.

Kurup R, Hunjan GS (2010) Intestinal parasites in St Lucia: a retrospective, laboratory-based study. Journal of Rural and Tropical Public Health 9: 24-30.

Kyrönseppä HJP, Goldsmid JM (1978) Studies on the intestinal parasites in African patients in Owamboland, South West Africa. Transactions of the Royal Society of Tropical Medicine and Hygiene 72: 16-21.

Laamrani H, Mahjour J, Madsen H, Khallaayoune K, Gryseels B (2000) *Schistosoma haematobium* in Morocco: Moving from control to elimination. Parasitology Today 16: 257-260.

Labbo R, Garba a, Louboutin-Croc JP, Ernould JC, Sellin B, et al. (2003) The spread of *Biomphalaria pfeifferi* in the Niger River valley, Niger. Annals of tropical medicine and parasitology 97: 209-212.

Lai Y-S, Biedermann P, Ekpo UF, Garba A, Mathieu E, et al. (2015) Spatial distribution of schistosomiasis and treatment needs in sub-Saharan Africa: A systematic review and geostatistical analysis. The Lancet Infectious Diseases 15: 927-940.

Lee H, Wykoff D, Beaver P (1966) Two cases of human schistosomiasis in new localities in Thailand. The American Journal of Tropical Medicine and Hygiene 15: 303-306.

Lee YH, Jeong HG, Kong WH, Lee SH, Cho HI, et al. (2015) Reduction of urogenital schistosomiasis with an integrated control project in Sudan. Plos Neglected Tropical Diseases 9: e3423.

Leonardo L, Rivera P, Saniel O, Villacorte E, Lebanan MA, et al. (2012) A national baseline prevalence survey of schistosomiasis in the Philippines using stratified two-step systematic cluster sampling design. Journal of Tropical Medicine 2012: 936128.

Leonardo LR, Acosta LP, Olveda RM, Aligui GDL (2002) Difficulties and strategies in the control of schistosomiasis in the Philippines. Acta Tropica 82: 295-299.

Leong S, Murugasu R, Chong K (1975) Schistosomiasis in the Orang Asli (a report of 9 cases). In: Nambiar RM, editor. Proceedings of the Tenth Malaysian Singapore Congress of Medicine: Academy of Medicine. pp. 184-186.

Leslie J, Garba A, Oliva EB, Barkire A, Tinni AA, et al. (2011) Schistosomiasis and soil-transmitted helminth control in Niger: cost effectiveness of school based and community distributed mass drug administration [corrected]. PLoS Neglected Tropical Diseases 5: e1326.

Liao CW, Sukati H, Nara T, Tsubouchi A, Chou CM, et al. (2011) Prevalence of *Schistosoma haematobium* infection among schoolchildren in remote areas devoid of sanitation in Northwestern Swaziland, Southern Africa. Japanese Journal of Infectious Diseases 64: 322-326.

Linehan M, Hanson C, Weaver A, Baker M, Kabore A, et al. (2011) Integrated implementation of programs targeting neglected tropical diseases through preventive chemotherapy: proving the feasibility at national scale. The American Journal of Tropical Medicine and Hygiene 84: 5-14.

Locketz L (1976) Health education in rural Surinam: use of videotape in a national campaign against schistosomiasis. Bulletin of the Pan American Health Organization 10: 219-226.

Lotfy WM (2009) Human schistosomiasis in Egypt: Historical review, assessment of the current picture and prediction of the future trends. Journal of the Medical Research Institute 30: 1-7.

Lotfy WM, Alsaqabi SM (2010) Human schistosomiasis in the Kingdom of Saudi Arabia: A review. Journal of the Medical Research Institute 31: 1-6.

Lyons GRL (1978) Assignment report: The Schistosomiasis Control Project at Raqqa, Syrian Arab Republic. At Raqqa, Syrian Arab Republic: World Health Organization: Regional Office for the Eastern Mediterranean.

Madsen H, Bloch P, Makaula P, Phiri H, Furu P, et al. (2011) Schistosomiasis in Lake Malaŵi villages. EcoHealth 8: 163-176.

Magnussen P (2003) Treatment and re-treatment strategies for schistosomiasis control in different epidemiological settings: a review of 10 years' experiences. Acta Trop 86: 243-254.

Mahmoud AaF (2004) Schistosomiasis (bilharziasis): From antiquity to the present. Infectious Disease Clinics of North America 18: 207-218.

Malek E (1985) Evaluation of schistosomiasis activities in the Hashemite Kindgdom of Jordan. U.S. Agency for International Development.

Massoud J, Arfaa F, Farahmandian I, Ardalan A, Mansoorian A (1982) Progress in the national schistosomiasis control programme of Iran. Bulletin of the World Health Organization 60: 577-582.

Mayor A (2014) Alexander the Great: A questionable death. History of Toxicology and Environmental Health: 52-59.

McCullough FS (1964) Observations on bilharziasis and the potential snail hosts in the Republic of the Congo (Brazzaville). Bulletin of the World Health Organization 30: 375-388.

Mccullough FS, Gayral PH, Duncan J, Christie JD (1980) Molluscicides in schistosomiasis control. Bulletin of the World Health Organization 58: 681-689.

McMullen DB (1968) Discussion of the paper by Willard H. Wright: "Schistosomiasis as a world problem". Bulletin of the New York Academy of Medicine 44: 3-6.

Meira MTV (1958) A Missao Permanente de Estudo t Combate de Endemias de Cabo Verde. Anais do Instituto de Medicina Tropical 15: 415-422.

Midzi N, Mduluza T, Chimbari MJ, Tshuma C, Charimari L, et al. (2014) Distribution of schistosomiasis and soil transmitted helminthiasis in Zimbabwe: Towards a national plan of action for control and elimination. PLoS Neglected Tropical Diseases 8: e3014.

Mills EA, Machattie C, Chadwick CR (1936) *Schistosoma haematobium* and its life cycle in Iraq. Transactions of the Royal Society of Tropical Medicine and Hygiene 30: 317-334.

Mkoji GM, Hofkin BV, Kuris AM, Stewart-Oaten A, Mungai BN, et al. (1999) Impact of the crayfish *Procambarus clarkii* on *Schistosoma haematobium* transmission in Kenya. American Journal of Tropical Medicine and Hygiene 61: 751-759.

Mohaddes K, Pesaran MH (2013) One hundred years of oil income and the Iranian economy: a curse or a blessing? Munich, Germany: CESifo Group.

Mohammed Ka, Haji HJ, Gabrielli A-F, Mubila L, Biswas G, et al. (2008) Triple co-administration of ivermectin, albendazole and praziquantel in zanzibar: a safety study. PLoS neglected tropical diseases 2: e171.

Mohan PC (2006) Madagascar: Rural Water Supply and Sanitation Project. Washington, DC: World Bank.

Mombeni H, Kheradmand A (2005) Schistosoma haematobium control in Khoozestan Province in Iran: prosperities and failures. Medical Journal of the Islamic Republic of Iran 19: 19-22.

Moné H, Ibikounlé M, Massougbodji A, Mouahid G (2010) Human Schistosomiasis in the Economic Community of West African States. Advances in Parasitology. pp. 33-91.

Mouchet F, Vera C, Bremond P, Devidas A, Sellin B (1990) [Urinary schistosomiasis in the Saharan mountain plateau of Air (Republic of Niger)]. Bull Soc Pathol Exot 83: 249-256.

Murugasu R, Dissanaike A (1973) First case of schistosomiasis in Malaysia. Transactions of the Royal Society of Tropical Medicine and Hygiene 67: 880.

Murugasu R, Wang F, Dissanaike A (1978) Schistosoma japonicum-type infection in Malaysia - report of the first living case. Transactions of the Royal Society of Tropical Medicine and Hygiene 72: 389-391.

Mwanakasale V, Siziya S, Mwansa J, Koukounari A, Fenwick A (2009) Impact of iron supplementation on schistosomiasis control in Zambian school children in a highly endemic area. Malawi Medical Journal 21: 12-18.

Naus CWa, Jones FM, Satti MZ, Joseph S, Riley EM, et al. (2003) Serological responses among individuals in areas where both schistosomiasis and malaria are endemic: cross-reactivity between Schistosoma mansoni and Plasmodium falciparum. The Journal of infectious diseases 187: 1272-1282.

Ndayishimiye O, Ortu G, Soares Magalhaes RJ, Clements A, Willems J, et al. (2014) Control of neglected tropical diseases in Burundi: partnerships, achievements, challenges, and lessons learned after four years of programme implementation. PLoS Neglected Tropical Diseases 8: e2684.

Ng'andu NH, Nkowane BM, Watts TE (1991) The health status of rural primary schoolchildren in Central Zambia. J Trop Med Hyg 94: 169-174.

Ogden S, Gallo K, Davis S, McGuire C, Meyer E, et al. (2013) WASH and the Neglected Tropical Diseases: A Manual for WASH implementers: Benin

Ohmae H, Sinuon M, Kirinoki M, Matsumoto J, Chigusa Y, et al. (2004) Schistosomiasis mekongi: From discovery to control. Parasitology International. pp. 135-142.

Ollivier G, Brutus L, Cot M (1998) La schistosomose intestinale à *Schistosoma mansoni* à Madagascar: extension et focalisation de l’endémie. Parasitologie 1966: 1-5.

Olveda DU, Li Y, Olveda RM, Lam AK, McManus DP, et al. (2014) Bilharzia in the Philippines: past, present, and future. International journal of infectious diseases : IJID : official publication of the International Society for Infectious Diseases 18: 52-56.

Ootsburg BFJ (1972) Clinical trial with hycanthone in Schistosomiasis mansoni in Surinam. Tropical Geography and Medicine 24: 148-151.

Opisa S, Odiere MR, Jura WGZO, Karanja DMS, Mwinzi PNM (2011) Malacological survey and geographical distribution of vector snails for schistosomiasis within informal settlements of Kisumu City, western Kenya. Parasites & Vectors 4: 226.

Ouldabdallahi M, Ouldbezeid M, Diop C, Dem E, Lassana K (2010) Épidémiologie des bilharzioses humaines en Mauritanie. L’exemple de la rive droite du fleuve Sénégal. Epidemiology 103: 317-322.

PAHO. Epidemiological profiles of neglected diseases and other infections related to poverty in Latin America and the Caribbean.; 2009; Washington, DC. PAHO.

PAHO/WHO. Schistosomiasis in Suriname. PAHO/WHO Scientific and Technical Material; 2007; St. George, Grenada.

Pancera CF, Alves AL, Paschoalotti MA, Chieffi PP (1997) Effect of wide spectrum anti-helminthic drugs upon *Schistosoma mansoni* experimentally infected mice. Revista do Instituto de Medicina Tropical de Sao Paulo 39: 159-163.

Paraense WL (2001) The Schistosome Vectors in the Americas. Memorias do Instituto Oswaldo Cruz. pp. 7-16.

Pfluger W (1982) Introduction of *Biomphalaria glabrata* to Egypt and other African countries. Royal Society of Tropical Medicine and Hygiene 76: 567.

Phiri AM, Phiri IK, Chota A, Monrad J (2007) Trematode infections in freshwater snails and cattle from the Kafue wetlands of Zambia during a period of highest cattle-water contact. Journal of Helminthology 81: 85-92.

Pitchford RJ. Bilharziasis in Swaziland: Report on the Situation in March 1956; 1958. pp. 735-750.

Poda JN, Sorgho H, Dianou D, Sawadogo B, Kambou T, et al. (2001) Parasitological profile of urinary schistosomiasis of the Sourou hydroagricultural complex of Burkina Faso. Bulletin de la Societe de Pathologie Exotique (1990) 94: 21-24.

Poda JN, Traoré A, Sondo BK (2004) Schistosomiasis endemic in Burkina Faso. Bulletin de la Societe de pathologie exotique (1990) 97: 47-52.

Pointier J-p (2001) Invading freshwater snails and biological bontrol in Martinique Island, French West Indies. Memorias do Instituto Oswaldo Cruz 96: 67-74.

Pointier J-p, David P, Jarne P (2011) The biological control of the snail hosts of schistosomes: The role of competitor snails and biological invasions. In: Toledo R, Fried B, editors. Biomphalaria Snails and Larval Trematodes. New York, NY: Springer New York. pp. 215-238.

Pointier JP (1993) The introduction of *Melanoides tuberculata* (Mollusca: Thiaridae) to the island of Saint Lucia (West Indies) and its role in the decline of *Biomphalaria glabrata*, the snail intermediate host of *Schistosoma mansoni*. Acta Tropica 54: 13-18.

Pointier JP, Guyard A (1992) Biological control of the snail intermediate hosts of *Schistosoma mansoni* in Martinique, French West Indies. Tropical Medicine and Parasitology 43: 98-101.

Pointier JP, McCullough F (1989) Biological control of the snail hosts of *Schistosoma mansoni* in the Caribbean area using *Thiara* spp. Acta Tropica. pp. 147-155.

Pointier JP, Théron A (1995) Ecology and control of the snail intermediate hosts of trematodes in an heterogenous environment: the Biomphalaria glabrata model in the insular focus of. Research and Reviews in Parasitology 55: 121-133.

Pointier JPP, Jourdane J (2000) Biological control of the snail hosts of schistosomiasis in areas of low transmission: the example of the Caribbean area. Acta Tropica 77: 53-60.

Prentice MA (1980) Schistosomiasis and its intermediate hosts in the Lesser Antillean Islands of the Caribbean. Bull Pan Am Health Organ 14: 258-268.

Reich MR (1998) International strategies for tropical disease treatments: Experiences with praziquantel Geneva, Switzerland: World Health Organization.

Rey JL, Sellin B, Mouchet F, Sellin E, Simonkovich E, et al. (1983) [Urinary schistosomiasis. 1st trial of mass treatment in the field (Niger)]. 707-710 p.

Rey L, Hachicha MT, Bahri M, Nacef T, Fareh R, et al. (1982) Schistosomiasis in Tunisia. Results after 10 years of the endemics control. Bulletin de la Societe de Pathologie Exotique et de Ses Filiales 75: 505-522.

Rollinson D, Knopp S, Levitz S, Stothard JR, Tchuem Tchuenté LA, et al. (2013) Time to set the agenda for schistosomiasis elimination. Acta Tropica 128: 423-440.

Romero R, Corachan M, Luis M (1989) Schistosomiasis in Sao Tome. A pilot study. Transactions of the Royal Society of Tropical Medicine and Hygiene 83: 81-82.

Rosa FM, Simoes M (1998) On the presence of trematode Schistosoma bovis (Sonsino, 1876) from Santiago Island. Garcia de Orta Serie de Zoologia 22: 69-72.

Rosenfield PL (1975) Development and verification of a schistosomiasis transmission model; with data from Bilharziasis Control Project and Dez Pilot Irrigation Project, Khuzestan Prov., Iran.

Ross AGP, Olveda RM, Acosta L, Harn Da, Chy D, et al. (2013) Road to the elimination of schistosomiasis from Asia: the journey is far from over. Microbes and infection / Institut Pasteur 15: 858-865.

Ruxin J, Negin J (2012) Removing the neglect from neglected tropical diseases: the Rwandan experience 2008-2010. Glob Public Health 7: 812-822.

Sagin DD, Ismail G, Fui JNF, Jok JJ (2001) Schistosomiasis malayensis-like infection among the Penan and other interior tribes (Orang Ulu) in upper Rejang River Basin Sarawak Malaysia. Southeast Asian Journal of Tropical Medicine and Public Health 32: 27-32.

Salami AO, Stampini M, Kamara AB, Sullivan Ca, Namara R (2014) Development aid and access to water and sanitation in Sub-Saharan Africa. Water International 39: 294-314.

Saliba EK, Abdel-Hafez SK, Tawfiq MR (1986) Schistosomiasis in Jordan : An unwelcomed guest. Parasitology Today 2: 91-93.

Saliba EK, Tawfiq MR, Kharabsheh S, Rahamneh J (1997) Urinary schistosomiasis contracted from an irrigation pool in Ramah, the southern Jordan Valley, Jordan. American Journal of Tropical Medicine and Hygiene 57: 158-161.

Samuels AM, Matey E, Mwinzi PNM, Wiegand RE, Muchiri G, et al. (2012) *Schistosoma mansoni* morbidity among school-aged children: A SCORE Project in Kenya. American Journal of Tropical Medicine and Hygiene 87: 874-882.

Sandbach FR (1976) The history of schistosomiasis research and policy for its control. Med Hist 20: 259-275.

Satrija F, Ridwan Y, Jastal, Samarang, Rauf A (2015) Current status of schistosomiasis in Indonesia. Acta Tropica. pp. 349-353.

Savioli L, Renganathan E, Montresor A, Davis A, Behbehani K (1997) Control of schistosomiasis - A global picture. Parasitology Today 13: 444-448.

Sawyer SG (2013) Schistosomiasis (Bilharzia) in Madagascar: A case study of a Neglected Tropical Disease. Independent Study Project (ISP) Collection.

Sayasone S, Mak TK, Vanmany M, Rasphone O, Vounatsou P, et al. (2011) Helminth and intestinal protozoa infections, multiparasitism and risk factors in Champasack Province, Lao People's Democratic Republic. PLoS Neglected Tropical Diseases 5: e1037.

Schneider CR, Hiatt RA, Malek EA, Ruiz-Tiben E (1985) Assessment of schistosomiasis in the Dominican Republic. Public Health Reports 100: 524-530.

Schneider MC, Aguilera XP, da Silva Junior JB, Ault SK, Najera P, et al. (2011) Elimination of neglected diseases in Latin America and the Caribbean: A mapping of selected diseases. PLoS Neglected Tropical Diseases 5: e964.

Schur N, Hürlimann E, Garba A, Traoré MS, Ndir O, et al. (2011) Geostatistical model-based estimates of schistosomiasis prevalence among individuals aged ≤20 years in West Africa. PLoS Neglected Tropical Diseases 5: e1194.

Schutte CH, Evans AC, Pammenter MD, Cooppan RM, Pretorius SJ, et al. (1995) Epidemiology and control of schistosomiasis mansoni in communities living on the Cuando River floodplain of East Caprivi, Namibia. Annals of Tropical Medicine and Parasitology 89: 631-644.

Schutte CHJ, VanDeventer JMG (1987) Schistosomiasis in eastern Caprivi. Part I. The prevalence of *Schistosoma* species and other infections in school children. Southern African Journal of Epidemiology and Infection 2: 71-75.

Schwarz E (1954) On African schistosomiasis. Transactions of the Royal Society of Tropical Medicine and Hygiene 48: 186-187.

Scott JA (1937) The incidence and distribution of the human schistosomes in Egypt. American Journal of Epidemiology 25: 566-614.

Senghor B (2010) Prévalence et intensiteé d'infestation de la bilharziose urogénitale chez des enfant d'âge ccolaire à Niakhar (milieu Rural Sénégalais) [D.E.A.]. Senegal: Thesis for the degree of D.E.A. Université Cheikh Anta Diop De Dakar.

Senghor B, Diallo A, Sylla SN, Doucouré S, Ndiath MO, et al. (2014) Prevalence and intensity of urinary schistosomiasis among school children in the district of Niakhar, region of Fatick, Senegal. Parasites & vectors 7: 5.

Sesay S, Paye J, Bah MS, McCarthy FM, Conteh A, et al. (2014) *Schistosoma mansoni* infection after three years of mass drug administration in Sierra Leone. Parasites & Vectors 7: 14.

Shamsedin EM (1961) The economic impact of development: a case of the Litani river and other projects in Lebanon. Gainsville, Florida: University of Florida. 83-118 p.

Shekhar K, Pathmanathan R (1987) Schistosomiasis in Malaysia. Review of Infectious Diseases 9: 1026-1037.

Simarrow PP, Ndong P (1996) Epidemiology and control of schistomosiasis in Equatorial Guinea. Research and Reviews in Parasitology 26: 137-140.

Simoes M, Rosa FM, Ferreira ML (2002) Experimental infection of some species of molluscs with Schistosoma bovis (strain of Santiago/Cape Verde). Garcia de Orta 24.

Sinuon M, Tsuyuoka R, Socheat D, Odermatt P, Ohmae H, et al. (2007) Control of *Schistosoma mekongi* in Cambodia: results of eight years of control activities in the two endemic provinces. Transactions of the Royal Society of Tropical Medicine and Hygiene 101: 34-39.

Siziya S, Mushanga M (1996) Importance of schistosomiasis in the Isoka district of Zambia: a prerequisite for its control using community participation. Soc Sci Med 42: 431-435.

Sodeman WAJ. *Thiara* (*Tarebia*) *granifera* (Lamarck): An agent For biological control of *Biomphalaria*; 1991 August 6-10; Manila, Phillippines. National Research Council Board on Science and Technology for International Development.

Sonsino (1893) Foreign University Intelligence. In: Wakley TH, editor. The Lancet vol 2. London: The Registered Proprietors, at the offices of "The Lancet". pp. 454.

Sornmani S (1976) Current status of schistosomiasis in Laos, Thailand and Malaysia. The Southeast Asian Journal of Tropical Medicine and Public Health 7: 149-154.

Sousa-Figueiredo JC, Stanton MC, Katokele S, Arinaitwe M, Adriko M, et al. (2015) Mapping of schistosomiasis and soil-transmitted helminths in Namibia: The first large-scale protocol to formally include rapid diagnostic tests. PLoS neglected tropical diseases 9: e0003831.

Southgate BA, Yacoub A (1987) The epidemiology of schistosomiasis in the later stages of a control programme based on chemotherapy: the Basrah study. 3. Antibody distributions and the use of age catalytic models and log-probit analysis in seroepidemiology. Transactions of the Royal Society of Tropical Medicine and Hygiene 81: 468-475.

Southgate V, Rollinson D, Kaukasl A, Almeda J, Sousa A, et al. (1994) Schistosomiasis in the Republic of Sao Tome and Principe: Characterization of *Schistosoma intercaiatum*. Transactions of the Royal Society of Tropical Medicine and Hygiene 88: 479-486.

Stauffer JR, Arnegard ME, Cetron M, Sullivan JJ, Chitsulo LA, et al. (1997) Controlling vectors and hosts of parasitic diseases using fishes. Bioscience 47: 41-49.

Stauffer JR, Madsen H, McKaye K, Konings A, Bloch P, et al. (2006) Schistosomiasis in Lake Malawi: Relationship of fish and intermediate host density to prevalence of human infection. EcoHealth 3: 22-27.

Steinmann P, Keiser J, Bos R, Tanner M, Utzinger J (2006) Schistosomiasis and water resources development: Systematic review, meta-analysis, and estimates of people at risk. The Lancet Infectious Diseases 6: 411-425.

Stich AHR, Biays S, Odermatt P, Men C, Saem C, et al. (1999) Foci of schistosomiasis mekongi, Northern Cambodia: II. Distribution of infection and morbidity. Tropical Medicine and International Health 4: 674-685.

Stoll NR (1999) This Wormy World: Address of the President, American Society of Parasitologists, December 27, 1946. The Journal of Parasitology 85: 392-396.

Stothard JR, Bremond P, Andriamaro L, Sellin B, Sellin E, et al. (2001) *Bulinus* species on Madagascar: Molecular evolution, genetic markers and compatibility with *Schistosoma haematobium*. Parasitology 123: S261-S275.

Stothard JR, French MD, Khamis IS, Basáñez M-G, Rollinson D (2009) The epidemiology and control of urinary schistosomiasis and soil-transmitted helminthiasis in schoolchildren on Unguja Island, Zanzibar. Transactions of the Royal Society of Tropical Medicine and Hygiene 103: 1031-1044.

Stothard JR, Loxton NJ, Rollinson D (2002) Freshwater snails on Mafia Island, Tanzania with special emphasis upon the genus *Bulinus* (Gastropoda : Planorbidae). Journal of Zoology 257: 353-364.

Subhani F, Nizamuddin R, Qasim A, Idrees N, Ahmed I, et al. (2014) The emerging threat of schistosomiasis spread in Pakistan. Tropical Biomedicine 31: 118-121.

Sukwa TY (1993) A community-based randomized trial of praziquantel to control schistosomiasis morbidity in schoolchildren in Zambia. Ann Trop Med Parasitol 87: 185-194.

Sukwa TY, Bulsara MK, Wurapa FK (1986) The relationship between morbidity and intensity of Schistosoma mansoni infection in a rural Zambian community. Int J Epidemiol 15: 248-251.

Sukwa TY, Bulsara MK, Wurapa FK (1987) Reduction in prevalence, intensity of infection and morbidity due to Schistosoma mansoni infection in a community following treatment with praziquantel. J Trop Med Hyg 90: 205-211.

Talla I, Kongs A, Verlé P, Belot J, Sarr S, et al. (1990) Outbreak of intestinal schistosomiasis in the Senegal River Basin. Annales de la Societe Belge de Medecine Tropicale 70: 173-180.

Tallo V, Carabin H, Alday P, Balolong E, Olveda R, et al. (2008) Is mass treatment the appropriate schistosomiasis elimination strategy? Bulletin of the World Health Organization 86: 765-771.

Tan M, Kusriastuti R, Savioli L, Hotez PJ (2014) Indonesia: An emerging market economy beset by Neglected Tropical Diseases (NTDs). PLoS neglected tropical diseases 8: e2449.

Tanaka H, Tsuji M (1997) From discovery to eradication of schistosomiasis in Japan: 1847-1996. International Journal for Parasitology 27: 1465-1480.

Tchuente LA, Ngassam R, Sumo L, Ngassam P, Noumendem C, et al. (2013) Mapping of schistosomiasis and soil-transmitted helminthiasis in the regions of Centre, East and West Cameroon. PLoS Neglected Tropical Diseases 6: e1553.

Temcharoen P, Viboolyavatana J, Tongkoom B, Sumethanurugkul P, Keittivuti B, et al. (1979) A survey on intestinal parasitic infections in Laotian refugees at Ubon Province, Northeastern Thailand, with special reference to schistosomiasis. The Southeast Asian Journal of Tropical Medicine and Public Health 10: 552-555.

Tikasingh ES, Wooding CD, Long E, Lee CP, Edwards C (1982) The presence of *Schistosoma mansoni* in Montserrat Leeward Islands. The Journal of Tropical Medicine and Hygiene 85: 41-43.

Touré S, Zhang Y, Bosqué-oliva E, Ky C, Ouedraogo A, et al. (2008) Two-year impact of single praziquantel treatment on infection in the national control programme on schistosomiasis in Burkina Faso. Bulletin of the World Health Organization 86: 780-788.

Urbani C, Sinoun M, Socheat D, Pholsena K, Strandgaard H, et al. (2002) Epidemiology and control of mekongi schistosomiasis. Acta Tropica. pp. 157-168.

Urbani C, Touré A, Hamed AO, Albonico M, Kane I, et al. (1997) [Intestinal parasitic infections and schistosomiasis in the valley of the Senegal river in the Islamic Republic of Mauritania]. Medecine tropicale : Revue du Corps de Sante Colonial 57: 157-160.

Utroska J, Chen M, Dixon H, Yoon S, Helling-Borda M, et al. (1989) An estimate of global needs for praziquantel within schistosomiasis control programmes. Geneva, Switzerland: World Health Organization. WHO/SCHISTO/89.102 Rev. 1 WHO/SCHISTO/89.102 Rev. 1. 1-93 p.

Utzinger J, Raso G, Brooker S, De Savigny D, Tanner M, et al. (2009) Schistosomiasis and neglected tropical diseases: towards integrated and sustainable control and a word of caution. Parasitology 136: 1859-1874.

Vaillancourt D, Ndiaye M (2008) Project performance assessment report, Arab Republic of Egypt, National Schistosomiasis Control Project. Washington D.C.: World Bank. 44466 44466.

Van Der Kuyp E (1969) Schistosomiasis mansoni in the Saramacca District of Surinam. Tropical Geography and Medicine 21: 88-92.

Vargas M, Malek EA, Perez JG (1990) Schistosomiasis mansoni in the Dominican Republic; prevalence and intensity in various urban and rural communities, 1982-198. Tropical Medicine and Parasitology 41: 415-418.

Vaughn CM, Olivier L, Hendricks JR, Mackie TT (1952) Mollusciciding operations in an endemic area of schistosomiasis in the Dominican Republic. The American Journal of Tropical Medicine and Hygiene 3: 518-528.

Wang L-d, Chen H-g, Guo J-g, Zeng X-j, Hong X-l, et al. (2009) A strategy to control transmission of *Schistosoma japonicum* in China. The New England Journal of Medicine 360: 121-128.

Watson JM (1953) Bilharziasis in South Persia. Royal Society of Tropical Medicine and Hygiene 47: 49-55.

Webster B, Southgate V, Littlewood D (2006) A revision of the interrelationships of *Schistosoma* including the recently described *Schistosoma guineensis*. International Journal for Parasitology 36: 947-955.

WHO (1971) Fourth Report on the World Health Situation 1965-1968. Geneva, Switzerland: World Health Organization. 340 p.

WHO (1983) Integrated vector control: Seventh report of the WHO Expert Committee on vector biology and control. Geneva, Switzerland: World Health Organization. 72 p.

WHO (1985) The control of schistosomiasis. Geneva, Switzerland: World Health Organization.

WHO (1993) The Control of Schistosomiasis: Second report of the WHO Expert Committee. Geneva, Switzerland: World Health Organization. 92 4 120830 9 92 4 120830 9.

WHO (1998) Fifty years of the World Health Organization in the Western Pacific Region, 1948-1998: Report of the Regional Director to the Regional Committee for the Western Pacific. Forty-ninth session. Manila, Philippines: World Health Organization Regional Office for the Western Pacific. 92 9061 122 7 92 9061 122 7.

WHO (1999) Schistosomiasis and intestinal parasites control. . Geneva, Switzerland: World Health Organization. 1-45 p.

WHO (2001) Report of the WHO Informal Consultation on Schistosomiasis in Low Transmission Areas: Control Strategies and Critera for Elimination. Geneva, Switzerland: World Health Organization.

WHO (2004) Health Systems Profile of the United Republic of Tanzania. Brazaville, Republic of Congo: World Health Organization Country Office for Africa.

WHO (2007) Inter-country meeting on strategies to eliminate schistosomiasis from the Eastern Mediterranean Region. Geneva, Switzerland: World Health Organization.

WHO (2007) Report of the global partners' meeting on Neglected Tropical Disease: A turning point 2007. Geneva, Switzerland: World Health Organization.

WHO (2008) Action Against Worms: Special Issue on Madagascar. Geneva, Switzerland: World Health Organization.

WHO. Elimination of schistosomiasis from low-transmission areas: Report of a WHO Informal Consultation; 2009; Bahia, Brazil. World Health Organization.

WHO (2009) WHO Country Cooperation Strategy: Sierra Leone. Brazzaville, Republic of Congo: World Health Organization Regional Office for Africa. 1-53 p.

WHO (2010) Commicable disease epidemiological profile: Cote d'Ivoire. Geneva, Switzerland: World Health Organization.

WHO (2010) Preventative chemotherapy and transmission control: Country Profile: Chad. Geneva, Switzerland: World Health Organization. 1-6 p.

WHO (2010) Preventative chemotherapy and transmission control: Country Profile: Rwanda. Geneva, Switzerland: World Health Organization.

WHO (2010) Preventive chemotherapy and transmission control: Country profile: Sierra Leone. Geneva, Switzerland: World Health Organization. 1-6 p.

WHO (2013) Somalia: WHO statistical profile. Geneva, Switzerland: World Health Organization.

WHO (2013) WHO schistosomiasis progress report 2001–2011 and strategic plan 2012–2020. Geneva, Switzerland: World Health Organization. WHO/HTM/NTD/PCT/2013.2 WHO/HTM/NTD/PCT/2013.2.

WHO. PCT Databank: Schistosomiasis. 2015 [cited 2015 Dec 31]; Available from: <http://www.who.int/neglected_diseases/preventive_chemotherapy/sch/en/>.

WHO/USAID (2010) Neglected Tropical Diseases in Indonesia: An integrated plan of action Ministry of Health Indonesia 2011-2015. WHO/USAID.

Wiwanitkit V (2005) Overview of clinical reports on urinary schistosomiasis in the tropical Asia. Pakistan Journal of Medical Sciences 21: 499-501.

Wolff T, Malewezi JG (1989) Organization and decentralization of the Malawi National Bilharzia Control Programme. Tropical Medicine and Parasitology 40: 201-204.

Wright WH (1947) Studies on schistosomiasis: The geographic distribution and molluscan intermediate hosts of the schistosomes maturing in man. National Institute of Health Bulletin 189: 1-48.

Wright WH (1968) Schistosomiasis as a world problem. Bulletin of the New York Academy of Medicine 44: 301-312.

Xianyi C, Liying W, Jiming C, Xiaonong Z, Jiang Z, et al. (2005) Policy and practice schistosomiasis control in China: The impact of a 10-year World Bank Loan Project (1992 – 2001). Bulletin of the World Health Organization 83: 43-48.

Yacoub A, Southgate BA, Lillywhite JE (1987) The epidemiology of schistosomiasis in the later stages of a control programme based on chemotherapy: the Basrah study. 2. The serological profile and the validity of the ELISA in seroepidemiological studies. Transactions of the Royal Society of Tropical Medicine and Hygiene 81: 460-467.

Youssef AR, Cannon JM, Al Juburi AZ, Cockett aTK (1998) Schistosomiasis in Saudi Arabia, Egypt, and Iraq. Urology 51: 170-174.

Zhou X-n, Bergquist R, Leonardo L, Olveda R (2008) Schistosomiasis: The disease and its control. 1-23 p.

Zhou X-N, Wang L-Y, Chen M-G, Wu X-H, Jiang Q-W, et al. (2005) The public health significance and control of schistosomiasis in China--then and now. Acta tropica 96: 97-105.
