# Supplementary material for: Global Assessment of Schistosomiasis Control Over the Past Century Shows Targeting the Snail Intermediate Host Works Best
Source: PLoS Negl Trop Dis. 2016 Jul 21;10(7):e0004794. doi: 10.1371/journal.pntd.0004794 (PMC4956325; doi:10.1371/journal.pntd.0004794)
Supplement: S2 Appendix — Detailed examples of cryptic social, ecological, and political factors: species invasions, sanitation, and ecosystem change. (DOCX) [file pntd.0004794.s002.docx]

**S2 Appendix**: **Supporting Text**

***Detailed examples of cryptic social, ecological, and political factors: species invasions, sanitation, and ecosystem change***

*Species invasions*

Long-term schistosomiasis reductions sometimes follow biological introductions (S1 Fig). Most notably, control programs introduced snails that outcompete the snail hosts for schistosomes to islands such as St. Lucia, Martinique, and Puerto Rico, leading to either elimination or >98% reductions in schistosomiasis (Table 1, main text, [[1](#_ENREF_1)]). These competitor snail species spread on their own across the Caribbean and were associated with >98% reductions (or elimination) in places like Antigua, Venezuela, and Montserrat (Table 1, main text, [[1](#_ENREF_1)]). The Louisiana crayfish, *Procambarus clarkii*, was introduced as a food item to Egypt during the early 1980s and spread throughout the Nile Delta by the 1990s [[2](#_ENREF_2)]. Subsequently, Egypt, has achieved a 99% reduction in schistosomiasis prevalence since baseline estimates (Table 1, main text). Although praziquantel has received the credit for these prevalence reductions in Egypt, crayfish prey on and can extirpate snail hosts for schistosomes ([[3](#_ENREF_3)], S1 Fig), suggesting invasive crayfish are a cryptic factor that enhanced the MDA programs by reducing re-infection.

*Development of sanitation improvements for reasons other than schistosomiasis control*

Starting in 1999, Indonesia improved sanitation in the main schistosome-endemic area to help develop and protect a national park for tourism [[4](#_ENREF_4)]. That region has since seen a 99.5% reduction in schistosomiasis prevalence (Table 1, main text). Similarly, in Zanzibar, the 85% reductions in schistosome prevalence (Table 1, main text) can, in part, be attributed to widespread improvements in sanitation and greater access to clean water on the islands; for the most part, governments implemented sanitation for general benefits such as tourism and welfare [[5](#_ENREF_5)]. For example, on Unguja island, the population with access to safe and clean water increased from 11% in 2003 to 97% in 2009 in the north, 29% to 90% in the south and 60% to 96% in the west and urban area[[6](#_ENREF_6)].

*Ecosystem change: Dam building*

Whereas irrigated-agricultural development increased schistosomiasis transmission in Sub-Saharan Africa and the Americas [[7](#_ENREF_7)], the opposite might have occurred in Southeast Asia. Specifically, Thailand built many more large dams during the 20^th^ century compared with its poorer neighbors Laos and Cambodia [[8](#_ENREF_8)]. Ironically, Laos and Cambodia have sustained *S. mekongi* transmission, despite coordinated control efforts, while schistosomiasis disappeared from Thailand without any clear control program. One explanation is that the snail intermediate host for *Schistosoma mekongi*, *Neotricula aperta*, unlike other schistosome-transmitting snail species, prefers fast flowing, well-oxygenated waters and has been shown to decline with dams and irrigation schemes [[9](#_ENREF_9)].

References for supplemental text

1. Pointier J, David P, Jarne P (2011) The biological control of the snail hosts of schistosomes: the role of competitor snails and biological invasions. In: Toledo R, editor. Biomphalaria snails and larval trematodes. New York: Springer Science+Business Media, LLC.

2. Khalil M, Sleem SH (2011) Can the freshwater crayfish eradicate schistosomiasis in Egypt and Africa? Journal of American Science 7: 457-462.

3. Mkoji GM, Hofkin BV, Kuris AM, Stewart-Oaten A, Mungai BN, et al. (1999) Impact of the crayfish Procambarus clarkii on Schistosoma haematobium transmission in Kenya. American Journal of Tropical Medicine and Hygiene 61: 751-759.

4. Izhar A, Sinaga RM, Sudomo M, Wardiyo ND (2002) Recent situation of schistosomiasis in Indonesia. Acta Tropica 82: 283-288.

5. Knopp S, Stothard JR, Rollinson D, Mohammed KA, Khamis IS, et al. (2011) From morbidity control to transmission control: time to change tactics against helminths on Unguja Island, Zanzibar. Acta Trop.

6. Knopp S, Stothard JR, Rollinson D, Mohammed KA, Khamis IS, et al. (2013) From morbidity control to transmission control: time to change tactics against helminths on Unguja Island, Zanzibar. Acta Trop 128: 412-422.

7. Steinmann P, Keiser J, Bos R, Tanner M, Utzinger J (2006) Schistosomiasis and water resources development: systematic review, meta-analysis, and estimates of people at risk. Lancet Infect Dis 6: 411-425.

8. FAO (Accessed: March 2015) AQUASTAT: Geo-referenced dams databases. <http://www.fao.org/nr/water/aquastat/dams/index.stm:> Food and Agriculture Organization of the United Nations.

9. Atwood S, Upatham ES (2012) Observations on *Neotricula aperta* (Gastropoda: Pomatiopsidae) population densities in Thailand and central Laos: implications for the spread of Mekong schistosomiasis. Parasites & Vectors 5.
