# Supplementary material for: Global Assessment of Schistosomiasis Control Over the Past Century Shows Targeting the Snail Intermediate Host Works Best
Source: PLoS Negl Trop Dis. 2016 Jul 21;10(7):e0004794. doi: 10.1371/journal.pntd.0004794 (PMC4956325; doi:10.1371/journal.pntd.0004794)
Supplement: S1 Table — (DOCX) [file pntd.0004794.s003.docx]

Table S1: List of variables searched for and recorded (when available) to assemble the database analyzed in this paper.

| **Category** | **Variables and options** | **Definitions** |
| --- | --- | --- |
| Quantitative measures of schistosomiasis infection and risk | Prevalence | The number of human cases of schistosomiasis infection in a particular population represented as a proportion of that particular population infected at a given time |
|  | Number infected | The total number of human cases of schistosomiasis infection in a particular country/territory |
|  | Intensity | The mean number of parasites found in infected hosts in a population, using the number of eggs/sample as a proxy - an indicator estimating disease burden and morbidity. This indicator was found to have been very rarely reported at the country-scale. |
|  | Number exposed or at risk | Total number of people within a country or territory at risk for contracting schistosomiasis disease. |
|  | Proportion at risk | The total number of people within a country or territory at risk of contracting schistosomiasis divided by the total country/territory population in that year. |
| Success status | Successful | A country or territory in which a coordinated national-scale control program was applied and sustained disease control was achieved, resulting in disease elimination, eradication, or non-endemicity for schistosomiasis. |
|  | Not yet successful | A country or territory where schistosomiasis is endemic and/or autochthonous cases are reported; status given if the disease is almost eliminated, has seen major or minor resurgence in transmission, if control was only partially effective, or if control was at best minimal in extent |
|  | Fortuitous elimination | A country or territory in which no coordinated national-scale control program was applied and yet, sustained disease control was achieved, resulting in disease elimination, eradication, or non-endemicity for schistosomiasis |
|  | Minimal control | A country or territory where schistosomiasis is endemic and/or autochthonous cases are reported; status given if control was at best minimal in extent |
| Type and extent of control | MDA | Mass drug administration: a deliberate effort to reduce schistosomiasis prevalence, intensity, and disease-related morbidity through the administration of chemotherapeutic drugs as drug administrations to whole communities or subsets of them |
|  | Engineering | Engineering interventions carried out specifically to reduce the incidence of schistosomiasis and its prevalence, intensity, and disease-related morbidity at a national level. Examples include sanitation improvements, building of bridges over infected watercourses, and cementing snail-infested bodies of water |
|  | Snail Control | Mechanical, chemical, or biological control of snail vector populations in order to reduce schistosomiasis transmission to human populations at a national level. Efforts primarily rely on use of molluscicides or the introduction of competitor, non-susceptible, snail species |
|  | Extent of use of each control measure (1) MDA, (2) snail control, (3) engineering controls | Estimated coverage for (1) proportion of at-risk population receiving chemotherapy treatment through MDA's within a particular country or territory, (2) proportion of communities in areas endemic for schistosomiasis where engineering interventions have been instituted, or (3) proportion of area endemic for schistosomiasis where snail control has been executed within a particular country or territory |
| Schistosome species | *Schistosoma mansoni* | Schistosome species causing an intestinal form of the disease, found in Africa, the Middle East, the Caribbean, Brazil, Venezuela and Suriname |
|  | *S. haematobium* | Schistosome species causing the urinary form of the disease, widespread in Africa and the Middle East |
|  | *S. japonicum* | Schistosome species causing an intestinal form of the disease found in Japan, China, and the Philippines |
|  | *S. mekongi* | Schistosome species causing an intestinal form of the disease found in Cambodia and the Lao People’s Democratic Republic |
|  | *S. guineensis or S. intercalatum* | Schistosome species causing intestinal forms of the disease found in the rainforests of central Africa; the two species were previously undifferentiated, but now recognized as two distinct taxa |
|  | *Schistosoma malayensis* | Schistosome species causing an intestinal form of the disease found only in Malaysia; minimal public health concern |
|  | *SmSh* | Both *S. mansoni* and *S. haematobium* endemic |
|  | *SmShSg or SmShSi combination* | All *S. mansioni, S. haematobium,* and *S. guineensis/intercalatum* endemic |
| Snail species | *Biomphalaria* spp*.* | Natural intermediate host snail reservoir of *Schistosoma mansoni* |
|  | *Bulinus* spp. | Natural intermediate host snail reservoir of *Schistosoma haematobium* |
|  | *Oncomelania* spp. | Natural intermediate host snail reservoir of *Schistosoma japonicum,* distributed in Japan, China, the Philipinnes, and Indonesia |
|  | *Neotricula aperta* | Natural intermediate host snail reservoir of *Schistosoma mekongi,* distributed in Cambodia, Laos, and Thailand |
| Economic indicators | Per capita GDP | Measure of the relative wealth of a country determined by the gross domestic product, or total economic output of the country, divided by the total number of individuals in that country; data obtained from the Maddison Project database (http://www.ggdc.net/maddison) |
|  | Country rank score | Overall score of a country assigned by determining the rank of each country's per capita GDP as compared to all other countries in a given year and dividing by the total number of countries, normalized from 0 to 1 |
| Other | Reservoir animals | Any non-human definitive mammalian hosts known to harbor infection with *Schistosoma* species also transmissible to humans in a country or territory, contributing to the environmental disease reservoir |
|  | Island? | A designation as an island or mainland country/territory |
|  | Notes | Any other information encountered that was deemed potentially relevant to schistosomiasis control outcomes or resources for control, or political, economic, and ecological contexts in a region. |
