# Supplementary material for: Global Assessment of Schistosomiasis Control Over the Past Century Shows Targeting the Snail Intermediate Host Works Best
Source: PLoS Negl Trop Dis. 2016 Jul 21;10(7):e0004794. doi: 10.1371/journal.pntd.0004794 (PMC4956325; doi:10.1371/journal.pntd.0004794)
Supplement: S2 Table — (DOCX) [file pntd.0004794.s004.docx]

Table S2:

Variables included (and regression formulas used) in the binary and quantitative analyses.

| Analysis | Symbol | Description |
| --- | --- | --- |
|  | Elimination | A binary variable indicating whether or not a country/territory achieved elimination, eradication, or non-endemicity for schistosomiasis |
|  | Prevalence | Schistosomiasis country-/territory-wide prevalence in a given year |
|  | Country | Name of the country/territory |
|  | Pop | A continuous variable: Human schistosome-infected-population at baseline (log-transformed # of people infected with schistosomiasis country-/territory-wide) |
|  | Control? | A binary variable indicating whether or not a coordinated control program was applied in that country/territory |
|  | Length | A continuous variable: control-end year minus control-start year |
|  | Year | A continuous variable: year (centered on control-start year as:  year minus control start year) |
|  | Strategy | A factor describing dominant control strategies, with 4 levels: MDA, Snail control, Both, Low coverage (see main text for more details) |
|  | Engineering | A binary factor indicating the presence or absence of engineering controls within a program (e.g. sanitation improvement, building bridges, cementing canals, etc.) |
|  | GDP_2013_ | A country’s per capita gross domestic product divided by midyear population, reported by World Bank indicators for 2013 http://data.worldbank.org/indicator: “GDP per capita (current US$)” |
|  | GDP*_variable_* | Inflation-adjusted (and log transformed) per capita gross domestic product for each year in which a country had disease data (ranging from 1910 to 2010), as reported in The Maddison-Project, http://www.ggdc.net/maddison/maddison-project/home.htm, 2013 version. |
|  | Water_access _2012_ | The percentage of the rural population using an improved drinking water source (e.g. piped water on premises, public taps or standpipes, tube wells or boreholes, protected dug wells, protected springs, and rainwater collection) as reported by World Bank indicators for 2012 http://data.worldbank.org/indicator: “Improved water source, rural (% of rural population with access)” |
|  | Island | A binary variable indicating whether or not a country/territory is an island or mainland |
| Binary analysis: Logistic regression | Formula | Elimination ~ Control? + Pop + Island + GDP_2013_ + Water_access_2012_ |
| Quantita-tive anlaysis:  GLMM (binomial) | Formula | Prevalence ~ random[Country] + Year + Prevalence + Length + Strategy + Engineering + GDP*_variable_* + Water_access _2012_  + interaction [Prevalence:Year] + interaction[Length:Year] + interaction[Strategy:Year] + interaction[Engineering:Year] + interaction[GDP*_variable_*:Year] + interaction[Water_access _2012_:Year] |
