# Supplementary material for: Global Assessment of Schistosomiasis Control Over the Past Century Shows Targeting the Snail Intermediate Host Works Best
Source: PLoS Negl Trop Dis. 2016 Jul 21;10(7):e0004794. doi: 10.1371/journal.pntd.0004794 (PMC4956325; doi:10.1371/journal.pntd.0004794)
Supplement: S3 Table — “3way interactions” signify a Strategy*Year*Engineering term in the full-model. The other variables are as in S2 Table. Those models above the dotted line were deemed to fit the data best. Removing the 3way interactions with engineering controls did not substantially change the model fit to data, whereas removing the other variables decreased the fit (increased Akaike’s information criterion (AIC)) substantially. (DOCX) [file pntd.0004794.s005.docx]

Table S3. Model selection results leading to the final model discussed in the main text. “3way interactions” signify a Strategy*Year*Engineering term in the full-model. The other variables are as in Table S2. Those models above the dotted line were deemed to fit the data best. Removing “3way interactions” did not substantially change the model fit to data, whereas removing the other variables decreased the fit (increased Akaike’s Information Criterion (AIC)) substantially.

| Model | K | AICc | ΔAICc | Cum wts |
| --- | --- | --- | --- | --- |
| W/O 3way interactions^§^ | 21 | 5315 | 0 | 0.74 |
| Full model | 25 | 5317 | 2.09 | 0.99 |
| W/O Year*Length | 19 | 5355 | 39.9 | 1.00 |
| W/O Year*Water_2012_ | 19 | 5391 | 75.6 | 1.00 |
| W/O Year*Engineering | 19 | 5447 | 132.1 | 1.00 |
| W/O Year*Control? | 15 | 5604 | 288.7 | 1.00 |
| Null Model Year only | 3 | 6248 | 932.7 | 1.00 |

^§^final model chosen and presented in Table S2 and Table 3 in the main text
