# Supplementary material for: Global Assessment of Schistosomiasis Control Over the Past Century Shows Targeting the Snail Intermediate Host Works Best
Source: PLoS Negl Trop Dis. 2016 Jul 21;10(7):e0004794. doi: 10.1371/journal.pntd.0004794 (PMC4956325; doi:10.1371/journal.pntd.0004794)
Supplement: S1 Fig — The suspected time-course of biological invasions in these regions—with most invasions occurring just before or during periods of maximal prevalence reduction—suggests the plausibility that invasions may have influenced schistosomiasis control outcomes for many, if not all, of these case studies. (DOCX) [file pntd.0004794.s006.docx]

Figure S1: Progression of schistosomiasis prevalence reductions (black lines) and timing of alien species introductions (intentional or inadvertent) which led to biological invasions of competitor snails (solid arrows) in the Caribbean or crayfish (dashed arrow) in Egypt. The suspected time-course of biological invasions in these regions – with most invasions occurring just before or during periods of maximal prevalence reduction – suggests the plausibility that invasions may have influenced schistosomiasis control outcomes for many, if not all, of these case studies.
